# Elucidation of the Synergistic Interaction Between Bilirubin and Casein Protein: An Integrated Spectroscopy and Computational Approach

**DOI:** 10.1021/acs.biomac.5c00795

**Published:** 2025-07-23

**Authors:** Sudhanshu Sharma, Jyoti Vishwakarma, Jacek Czub, Subrahmanyam Sappati, Krishna Gavvala

**Affiliations:** † Department of Chemistry, 233600 Indian Institute of Technology Hyderabad, Kandi, Sangareddy, Telangana 502284, India; ‡ Department of Pharmaceutical Technology and Biochemistry, Gdańsk University of Technology, 80-233 Gdańsk, Poland; § Department of Physical Chemistry, Politechnika Gdanska, 80-233 Gdańsk, Poland; ∥ BioTechMed Center, Gdańsk University of Technology, 80-233 Gdańsk, Poland

## Abstract

Herein, we unveil the interaction between bilirubin (BIL), a liver metabolite, and a milk protein, casein (CAS), through an integrated experimental–computational approach. Encapsulation of BIL within CAS protein micelles was characterized by using UV–vis absorption, steady-state fluorescence, and circular dichroism (CD) spectroscopy. CD analysis revealed conformational modulation of BIL upon encapsulation, accompanied by Förster resonance energy transfer (FRET) from CAS’s tryptophans to BIL. ^1^H NMR measurements determined specific binding interactions of BIL functional groups involved in micellar interactions, correlating photophysical and electronic properties. The binding affinity of BIL in CAS micelles was found to be on the order of 10^4^ M^–1^ with a spontaneous binding process (−24.56 kJ/mol) driven by entropy gains (467.17 J/mol). TDDFT calculations unveiled red shifts in BIL’s absorption spectra caused by the protein environment. This integrated experimental–computational study provides novel insights into synergetic interactions and structural dynamics between BIL and CAS, shedding light on the influence of milk proteins on bilirubin’s behavior.

## Graphical abstract

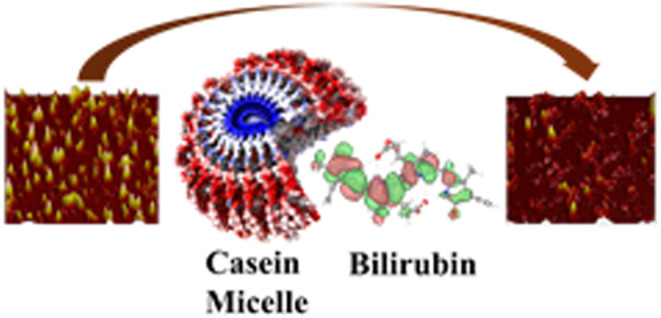

## 1 Introduction

Bilirubin (BIL) is the main pigment responsible for jaundice, and its level should not exceed a certain value to avoid critical conditions in adults and newborns.^1^ Jaundice or hyperbilirubinemia is highly dangerous for newborns as compared to that in adults, since at the onset of a baby’s life, the BIL level starts shooting up with time at a very high pace due to the limitation of an underdeveloped liver. Within a span of just 3–4 days from birth, the BIL level reaches its highest value compared to that in adults, which leads to neurological disorders in infants.^1,2^ Rising BIL levels can be sourced from various reasons such as pregnancy,^3^ liver diseases,^4^ increased red blood cell breakdown,^5^ and also herbal supplements.^6^ BIL is the breakdown product of the heme (ferroprotoporphyrin IX) ring when senescent red blood cells (RBCs) get digested by macrophages (immune cells) in the spleen and liver.^7^ Among the heme proteins, 80% of the BIL production is sourced from the heme rings of hemoglobin. Chemically, in heme, there are four pyrrole rings joined by four methene bridges (−CH =) in one plane. After the metabolic enzyme treatment from heme oxygenase and biliverdin reductase, BIL IXα is generated, which does not have an iron atom and a cyclic structure like heme. Although similar to heme, four chromophore rings are present in noncyclic BIL, but only the first two and the last two rings are in conjugation, with a saturated central methylene group (−CH_2_−) in between the pairs. This makes the two planes of chromophore pairs in the molecule exist at dihedral angles of 98–104°.^8^ Both the planes have their individual electric transition dipole moments that source the chirality in the BIL molecule. Due to this, there exist intramolecular exciton interactions between the two dipole moments, leading to M (negative helicity) and P (positive helicity) helical.^9^ Recent reports confirm that the milk protein conjugates have a significant effect on liver function.^10,11^ The proteins present in milk have been proven to regulate the BIL absorption in the intestine;^11^ however, the possible outcomes and mechanistic understanding of their interaction have not been explored. Being an important liver metabolite, the interaction study of BIL with biomolecules such as proteins forms the basis for significant therapeutic applications.

Casein (CAS) protein is one of the important milk proteins made up of three major proteins, namely, α-CAS, β-CAS, and κ-CAS, with an average molecular weight of 22.5 kDa. The major proteins involved in micelle formation are α- and β-CAS, whereas k-CAS resides on the micelle surface and stabilizes the micelle structure.^12^ The micelle formation by protein is itself a unique feature; however, the structure of CAS micelles is still under exploration and lacks a crystal structure. The reason for the different types of jaundice, such as neonatal and breast milk jaundice, is that the β-glucuronidase enzyme, present in milk, detaches glucuronic acid from conjugated (or direct) BIL, which elevates the levels of unconjugated (or indirect) BIL in jaundice.^13^ This unconjugated BIL is then readily absorbed by the intestine. Although this can be the potential reason for hyperbilirubinemia, an exact understanding of the reason is not clear. Being a major milk protein, CAS exists as self-assembled structures,^14^ known as protein-based micelles, with excellent ligand encapsulation ability. Understanding the interaction of the CAS micelles with BIL not only is an important fundamental research aspect but also can find potential biomedical applications in the diagnosis and treatment of jaundice.

We utilized multispectroscopic studies using intrinsic and extrinsic fluorophores to characterize the interactions and encapsulation of BIL with CAS. These include UV–vis absorption, steady-state fluorescence, synchronous fluorescence, time-resolved fluorescence, and circular dichroism measurements. To elucidate the interaction at a better resolution, a ^1^H nuclear magnetic resonance (NMR) study was conducted. Atomic force microscopy (AFM) was used to study the morphological changes of the surface of CAS micelles upon interacting with a high content of BIL. For molecular-level information, an MD simulation study was performed between β-CAS as a protein and BIL as a ligand. Further, to understand the spectroscopic aspects of the experimental results, we employed ab initio simulations and umbrella sampling simulations for binding energy calculations, followed by time-dependent density functional theory (TDDFT) for excited-state calculations.

## 2 Results and Discussion

### 2.1 Steady-State Absorption Measurements

The electronic properties of BIL are characteristically sensitive and significantly influence its bioactivity. Understanding the modulation in ground- and excited-state features when confined in a micelle assembled from a protein comprises diverse noncovalent interactions. UV–vis absorption and fluorescence spectroscopy have been a highly essential tool for characterizing a range of biomolecular interactions, and spectra were corrected by the inner filter effect (eq S1). The initial UV–vis spectra in Figure 1a show the concomitant absorption peak of the BIL molecule situated at 438 nm. As per the literature, for a molecule like BIL, the absorption maximum should be situated at 420 nm, which is 18 nm apart from the observed maxima of BIL.^15^ This is attributed to the presence of internal hydrogen bonds between carboxyl protons and pyrrole rings that repel the formation of the betaine structure of BIL.

**Figure 1:**
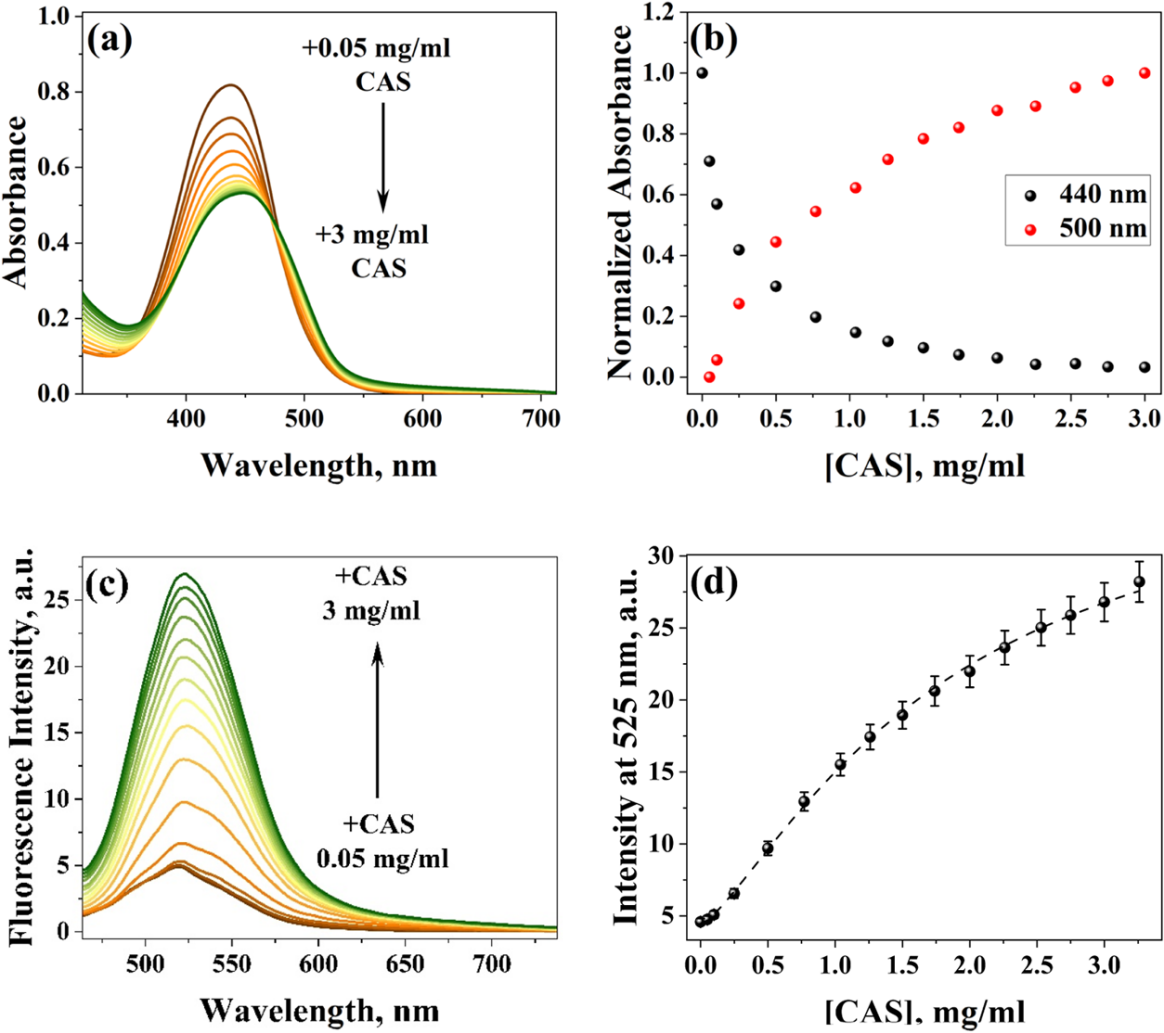
(a) UV–vis absorption spectra of BIL in the absence and presence of CAS (0.05 to 3 mg/mL). (b) Normalized absorbance at 440 and 500 nm as a function of CAS protein. (c) Emission spectra (λ_ex_ = 440 nm) of BIL (16.8 μM) in the absence and presence of CAS (0.05 to 3 mg/mL). (d) Fluorescence intensity of emission maxima of BIL as a function of CAS. Measurements are done in PB (pH = 7.3) at 298 K.

Upon gradual addition of CAS, the absorption maxima underwent a decline in their value starting from ∼0.8 until they reached saturation at a value of ∼0.5 of the absorbance. The decline in absorbance maxima to ∼0.5 occurs when the concentration of CAS protein reaches near ∼1.2–1.5 mg/mL, while an increase occurs at 500 nm (Figure 1b). This concentration range has been earlier reported to be the concentration (CMC) at which the CAS protein assembles to form micellar structures.^16^ Upon further addition of CAS above its CMC, the absorption maxima do not undergo any change, suggesting encapsulation of BIL inside the micelles. In addition to this, there is a red shift of 11 nm in BIL absorbance from 438 to 449 nm, as shown in the normalized plot (Figure S1a). The absorbance maxima wavelength, if plotted against the CAS concentration, can retrace the encapsulation of BIL in CAS micelles, due to the saturation near 1.8 mg/mL concentration of CAS (Figure S1b). This gives a picture of polarity changes in the vicinity of BIL molecules inside CAS micelles. Interestingly, the shift may also correspond to hydrogen bonding interactions between BIL and CAS^17,18^ and is further explored in computational studies in the later sections. As a control, the protein absorption before and after micelle formation remains situated in the region of 280 nm with no contribution in the absorption region of BIL, confirming the changes attributed to modulation in the electronic levels of BIL when encapsulated.

Additionally, the absorbance at 470 nm did not undergo any change in its value and can be considered as the isosbestic point shown at the intersection. The physical significance of this point indicates that the encapsulated BIL is in equilibrium with the free form in the solution. The absorption spectrum of free BIL has an fwhm of 95 nm. The fwhm of encapsulated BIL in the end absorption spectra is 117 nm, which is much higher than that of free BIL. Although the red shift of BIL absorbance indicates the environment polarity change, an increase in fwhm has been previously stated due to the association of more than one BIL molecule with each other.^19^ So, in this case, there is a possibility of more than one BIL molecule getting encapsulated inside the protein micelle.

To quantify the binding affinity between BIL and CAS, we used the Hill1 equation (eq S2). Figure S2 indicates the fitting (*R* ^2^ = 0.99) of BIL absorbance by the Hill1 equation. The binding constant was calculated by the reciprocal of k_D_ (1/0.75 = 1.33 (mg/mL)^−1^) and using the average molecular weight of CAS (22, 500 Da), the binding constant was found to be 3 × 10^4^ M^–1^, and the number of binding sites, n, is 1.32. This nanovalue may indicate the encapsulating stoichiometry to be more than one molecule of BIL, supporting the possibility of more than one molecule of BIL in CAS micelles.

### 2.2 Steady-State Fluorescence Measurements

BIL is known to have weak fluorescence characteristics when excited at its 440 nm absorbance maximum.^20^ Figure 1c illustrates emission spectra recorded at 440 nm excitation for BIL in the absence and presence of the CAS protein. On gradually increasing the CAS concentration, the fluorescence of BIL peaked at 525 nm and started becoming prominent. On recording the excitation spectra at 525 nm emission, in a similar manner, BIL initially had a weak, intense peak at ∼440 nm (Figure S3). On increasing the CAS concentration, the intensity of this peak increased prominently due to the encapsulation of BIL molecules in protein micelles. The blank spectra of CAS aggregates were checked, and no band was found in the mentioned scan range. On observing the spectral maxima position in the excitation spectra of the BIL–CAS complex, it lies within the range of 450–460 nm, which is similar to the red-shifted absorption maxima position given in an earlier section. This indicates that the source of emission at 525 nm for the excited-state CAS–BIL complex is from the same species that is present in the ground state.

Further, we have estimated the binding constant using the Hill1 equation (eq S3).^21^ Figure 1d indicates the fitting (*R* ^2^ = 0.99) of the binding curve, which gives a binding constant of the same order of 10^4^ from absorbance to be 1.08 × 10^4^ M^–1^. Also, similar to absorbance fitting, the number of binding sites is calculated to be 1.21, supporting more than one BIL molecule in the encapsulation of the CAS micelle.

### 2.3 Circular Dichroism Measurements

The CD spectrum of BIL complexes with a variety of serum proteins has been widely studied in the literature to investigate the different conformations of BIL achieved within the protein pockets.^22^ BIL is chiral in nature, which is sourced from the P and M helical enantiomers in its rigid-tile conformation.^23^ Because of these enantiomers, there is some weak CD signal near 420 nm (Figure 2a) that is near its absorption maxima (440 nm). On gradually adding CAS to BIL, interestingly, the 420 nm band undergoes a red shift. This red shift started to be observed at only 0.1 mg/mL CAS and continued until it saturated near the CMC of the CAS protein (0.8–1.5 mg/mL). This observed shift is near 43 nm from 420 to 463 nm wavelength minima. Blank CAS was checked, and no signal was found in the measured range. As mentioned, protein pockets can induce conformational changes in BIL. These changes suggest that there is a transformation in the chiral behavior when BIL is encapsulated inside the CAS protein. Since inside the protein micelle, BIL emits fluorescence at 525 nm on excitation at 440 nm, these chiral changes are associated with the photophysical characteristics of BIL and are responsible for the observed fluorescence enhancement and absorption changes.

**Figure 2:**
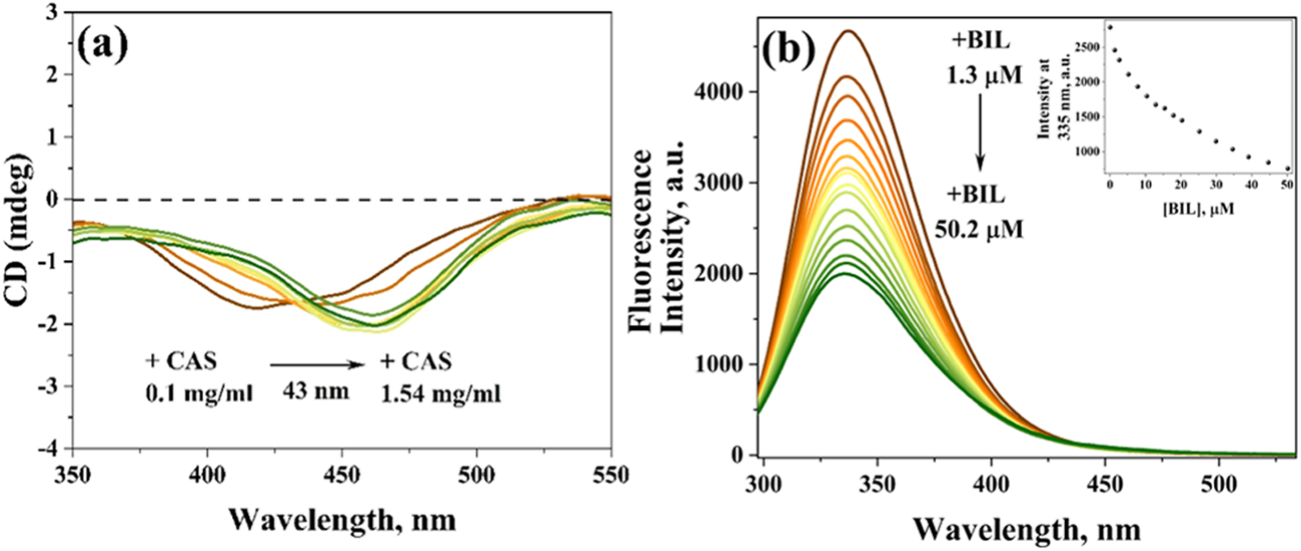
(a) CD spectra of BIL (20 μM) in the absence and presence of the CAS protein (0.1–1.54 mg/mL). (b) Emission spectra (λ_ex_ = 280 nm) of the CAS protein (2 mg/mL) in the absence and presence of BIL (1.3–50.2 μM). The inset represents emission intensity at wavelength maxima as a function of [BIL]. Measurements are done in PB (pH = 7.3) at 298 K.

### 2.4 Intrinsic Fluorescence Measurements

α-S1-CAS has two Trps at 164 and 109, whereas β-CAS and κ-CAS have one Trp each at 143 and 76 locations, respectively. The combined photophysical behavior of these amino acids can detect changes in the micellar structure of the CAS protein when BIL is introduced due to their locations in the hydrophobic region of micelles.^24^ Trp natural fluorescence in its free form is at 350 nm; however, it is evident from blue-shifted emission at 335 nm in CAS micelles that these are present in the hydrophobic micellar region of the CAS protein as reported earlier.^24^

Figure 2b (brown) indicates changes in the intrinsic fluorescence of Trp present in CAS micelles (2 mg/mL > CMC) when BIL is introduced. Upon adding even a small amount of BIL, there is a huge fluorescence quenching of CAS micelles, including the normal (∼17 μM) as well as abnormal (∼34 μM) human levels of BIL (adults are declared to have jaundice at 34 μM BIL;^25^ inset of Figure 2b). So, the quenching with even a small amount of BIL indicates the accessibility of BIL in the hydrophobic regions, where Trps are situated. However, there was no shift in the wavelength maxima of CAS, indicating that BIL does not induce a substantial change in the dielectric medium of Trps. A similar trend of quenching without wavelength shift is also observed in the excitation spectra shown in Figure S4.

Synchronous fluorescence was used to characterize interactions in the individual local vicinity of Trp and Tyr amino acids. Combinedly, α-S1, β-CAS, and κ-CAS contain 5 Trps and 23 Tyr amino acids. Figure S5a,b illustrates changes in the synchronous spectra of CAS micelles (2 mg/mL) upon introducing BIL. Similar to the steady-state emission results, the Trp spectra of CAS underwent large quenching upon adding BIL. This decrease is not associated with any shift in the wavelength (Figure S5c), which supports the earlier conclusion from fluorescence spectra that Trp location is in a similar environment after the conformational change of the micelles. But in the case of Tyr, along with a decrease in intensity, there is a noticeable blue shift of 3 nm (Figure S5d). These results indicate that the Tyr residues have moved to a more hydrophobic environment compared to that present in the native conformation of the CAS micelles.

So, the fluorescence quenching results indicate two mechanistic insights in the interaction of BIL with CAS micelles: first, there can be an excited-state energy transfer mechanism (explained in a later section) between Trp as the donor and BIL as the acceptor, which causes the quenching, and second, the structures of CAS micelles get deformed by BIL, causing shifts in the local dielectric media.

### 2.5 Determination of the Quenching Mechanism and Thermodynamic Parameters

Temperature-dependent fluorescence studies were employed to determine the quenching mechanism of the CAS micelles by BIL. Both *K*_a_ and *K*_sv_ can be calculated by the Stern–Volmer equation (eq S4). Figure S6a depicts the plot of ((F_0_–F)/F) versus [BIL] at three different temperatures (i.e., 293.15, 303.15, and 313.15 K). The values of *K*_sv_ and *k*_q_ are illustrated in Table 1 with rising temperature. The direct correlation of *K*_sv_ with temperature suggests that a dynamic quenching occurs between BIL and CAS. Moreover, the obtained value of *k*_q_ deciphers a higher order of bimolecular diffusion rate constant, 10^14^ M^–1^_S_^–1^ (more than the maximum scattering collision quenching constant, 2 × 1010 M^–1^ s^–1^), depicting that some of the quenching can be due to ground-state complex formation (static quenching).^26^ Hence, both static and dynamic quenching mechanisms are involved in CAS and BIL.^27^

**Table 1:** Stern–Volmer Fluorescence Quenching Constant and Thermodynamic Parameter of the CAS Protein (2 mg/mL) in the Absence and Presence of BIL (7-40 μM)^a^

*T* (K)	*K*_sv_ (× 10^3^ M^–1^)	*k*_q_ (× 10^13^ M^–1^ s^–1^)	*K*_a_ (× 10^5^ M^–1^)	Δ*H* (kJ/mol)	Δ*S* (J/mol)	Δ*G* (kJ/mol)
293.15	3.00	1.071	0.0431	117.06	467.17	–19.88
303.15	19.80	7.068	0.101			–24.56
313.15	67.56	24.12	1.16			–29.23

a Measurements are done in PB (pH = 7.3) at 293.15, 303.15, and 313.15 K.

Furthermore, the equilibrium between free and bound molecules, when the molecules bind independently to a set of equivalent sites on a macromolecule, can be estimated by the Hill equation (eq S5). The plot of the regression curve of Log ((F_0_–F)/F) versus Log [BIL] is shown in Figure S6b. The values evaluated for *K*_a_ are represented in Table 1, indicating that BIL binds to CAS with a higher binding affinity with an increase in temperature, which implies stable complex formation. The obtained values are in good agreement with the trend in *k*_sv_ and *k*_q_.

Essentially, the binding forces between any drug molecules were evaluated by thermodynamic parameters to determine the type of interactions. According to Ross and Subramanian^28^, both Δ*H* < 0 and Δ*S* < 0 values are attributed to van der Waals forces and hydrogen bonding, whereas Δ*H* > 0 and Δ*S* > 0 indicate hydrophobic interactions, and in the case of electrostatic interaction, Δ*H* < 0 and Δ*S* > 0. So, to determine the type of interaction between BIL and CAS, the van’t Hoff equation (eq S6) and the Gibbs free energy equation (eq S7) were utilized.

The van’t Hoff plot between *ln K*_a_ and 1/*T* was used to obtain Δ*H* and Δ*S* from the slope and intercept (Figure S7). The values of Δ*G*, Δ*H*, and Δ*S* for CAS and BIL are summarized in Table 1. Both positive values were obtained for Δ*H* and Δ*S* as well, depicting the role of hydrophobic forces in the binding process of CAS and BIL. This also quantifies the influence of BIL on the CAS micelles assembled from the hydrophobic forces between the proteins.

### 2.6 Time-Resolved Fluorescence Measurements

Using the time-correlated single-photon counting (TCSPC) technique in fluorescence measurements, the decay in population of the first singlet excited state can be estimated in terms of fluorescence photon counts. Figure S8a illustrates the fluorescence decay profiles of Trp in a CAS micelle (2 mg/mL > CMC) in the absence and presence of different quantities of BIL. It is observable from the decays that upon introducing BIL, the excited-state population of the CAS Trp takes less time to reach the electronic ground state. Since from thermodynamic measurements, dynamic quenching was determined, the current speeding up the population decays can be attributed to the collision of BIL with CAS micelles in the excited state. Upon fitting the decay profiles by a biexponential equation (eq S8), there are two lifetime components, 2.2 and 5.8 ns. These component values can be allotted due to conformational isomers of Trps.^29^

Broadly, there are two types of locations where Trp lies: one within the micellar region made from α-CAS and β-CAS, and another on κ-CAS, which stabilizes the surface of micelles. So, it is difficult to specify the lifetime component values, but it can be speculated that biexponential behavior is probably due to these two locations. On observing the behavior of these components, both lifetime components underwent a decrease in their value, but with a greater decrease of 0.9 ns in the 5.8 ns component (Table S1). Since BIL is getting encapsulated inside CAS micelles, the more change in the longer lifetime component can be attributed to the BIL interaction with the Trps of α-CAS and β-CAS inside the hydrophobic micelles, whereas the changes in the short component can be due to the interaction with the Trp of κ-CAS present on the surface. Figure S9 depicts the change in the pre-exponential factor of their respective fluorescence lifetimes on introducing BIL in CAS micelles. It is evident that the percentage of the short component increases according to the BIL concentration, and at the same time, that of the long component decreases. This leads to a decrease in the overall average lifetime (τ_av_) of the system. So, both hydrogen bonding interactions between BIL and micelles (interpreted from UV–vis results) and hydrophobic interactions (thermodynamic study) activate the nonradiative channels of tryptophan amino acids in the excited state. Due to this, the excited-state complex radiates back to the ground state in less time, leading to a decrease in fluorescence intensity. So, a decrease in τ_av_ supports the fact that the binding equilibria involve a dynamic mechanism of the interaction between BIL and CAS in the excited state. To calculate the dynamic quenching constant, we fitted Figure S8b by eq S9. So, *K*_*D*_ was determined to be 3.5 × 10^3^ M^–1^ for CAS–BIL complexation. Therefore, the quenching observed in steady-state studies has a dynamic quenching contribution in the excited state. The mechanism of quenching can be further elucidated with the help of FRET measurements.

### 2.7 Förster Resonance Energy Transfer Calculations

FRET is a robust technique for determining the mechanism of interaction and measuring the distance between two chromophores acting as the donor and acceptor of radiationless energy between them and has been accepted as a pivotal technique having applications in several areas of biophysics and biochemistry.^30^ CAS Trps emitted at 340 nm have a broad range of fluorescence until 450 nm. This obtains an overlapping region between the donor emission and acceptor (BIL) absorption (Figure S10). So, this confirms a potential FRET mechanism from CAS Trp to the BIL molecule in the micelle. The binding mode of Trp and BIL is also elucidated from the computational studies in the later section. Using eq S12, we calculated the overlap integral *J*(λ) to be 9.31 × 10^13^ nm^4^ M^–1^ cm^–1^. Keeping the respective values in eq S11, the Förster radius came to be equal to 33.82 Å. Using fluorescence quenching data in eq S10, the FRET efficiency was calculated as 57.19% from CAS Trp to BIL. Therefore, utilizing these values, the donor–acceptor distance was determined as 32.23 Å. We also calculated the rate of energy transfer (*k*_ET_) from CAS to BIL by using eq 1

1 $$k_{\mathrm{ET}}=\frac{1}{\tau_{D}}{\left(\frac{R_{0}}{r}\right)}^{6}\sec$$

where τ_*D*_ is the donor lifetime in the absence of an acceptor. *R*_0_ is the Förster radius, and *r* is the D–A calculated distance. It was found that the rate of FRET, which is a type of nonradiative process, is 3.74 × 10^8^ s^–1^. Using eq 2, we obtain the following

2 $$E=\frac{k_{\mathrm{ET}}}{k_{r}+k_{\mathrm{ET}}+\sum k_{\mathrm{nr}}}$$

where *E* is the FRET efficiency calculated from eq 1, *k*_r_ is the rate of radiative decay calculated from *k*_r_ = ϕ/τ_D–A_, and Σ*k*_nr_ is the summation of the rest of the nonradiative decay rates. *k*_r_ is calculated from the quenched quantum yield and fluorescence lifetime at 50.2 μM BIL and is determined as 1.4 × 10^8^ s^–1^. So, using *E*, *k*_ET_, and *k*_r_, the value Σ*k*_nr_ is estimated as 1.27 × 10^8^ s^–1^. It is to note that the Σ*k*_nr_ is without adding *k*_ET_, adding of which will make it 5.01 × 10^8^ s^–1^. So, *k*_nr_ is more than *k*_r_, which resulted in the observed fluorescence quenching. To elucidate deeper into the specific interactions of BIL with CAS, an NMR study was conducted.

### 2.8 ^1^H NMR Spectral Study

Figure 3 depicts the ^1^H NMR spectra (400 MHz, DMSO-*d*_6_) of BIL (10 mg/mL) in the absence and presence of CAS (1 mg/mL), suggesting the chemical shift variations and spectral perturbations, which reflect molecular interactions at the atomic level. In the absence of CAS (Figure 3a), BIL exhibits well-resolved signals in the aliphatic, aromatic, and exchangeable proton regions. The ^1^H peaks of the BIL were first characterized (Figure 3a) in DMSO-*d*_6_ solvent and verified with the earlier literature:^31^ δH = 11.92 ppm (2H, −COOH), δH = 9.93, 10.06, 10.46, 10.50 ppm (4H, –NH), δH = 5.29–6.87 ppm (8H, –vinyl), δH = 3.99 ppm (2H, −CH_2_ at the central carbon of BIL), δH = 2.40–2.52 ppm (8H, −CH_2_–CH_2_– protons adjacent to carboxyl groups), and δH = 1.91–2.17 ppm (12H, −CH_3_). These chemical shift values validate the chemical structure of BIL with 36H. Upon the addition of CAS (Figure 3b), significant spectral perturbations are observed across multiple regions, strongly suggesting a molecular interaction between them. To avoid spectral overlap, the concentration of CAS was optimized (1 mg/mL) and kept significantly lower than that of BIL (10 mg/mL). At this concentration, CAS signals were not visible in the ^1^H NMR spectra, as confirmed by control experiments (Figures S11–S13). Most notably, the amide –NH signals (δH = 9.93–10.5 ppm) exhibit signal attenuation, broadening, and deshielding, suggesting H-bond formation between the BIL and polar residues of CAS. Similarly, the carboxylic acid proton at δH ∼11.92 ppm shows clear signal attenuation, consistent with dynamic proton exchange or H-bonding interactions with CAS. The complete reduction of the signal complements the evidence of ligand encapsulation or CAS–BIL’s strong interaction, where the local environment restricts molecular tumbling. Since protein is a non-tumbling macromolecule, when a ligand is encapsulated or bound to it, it suppresses the tumbling motion of the ligand.^32,33^ This leads to fast transverse relaxation of the ^1^H protons of the BIL and causes the disappearance with signal broadening. These measurements give strong evidence that the encapsulation of BIL inside CAS micelles involves the disruption of the intermolecular H-bonding existing among the carboxyl and lactam (−NH) protons of BIL. The interaction with the CAS micelle led the molecule to change the molecular coplanarity, which led to the disappearance of the signals. Hence, these chemical shift perturbations, signal broadening, and disappearance of key exchangeable protons give strong evidence about the encapsulation of BIL inside CAS micelles. The use of DMSO-*d*_6_ could be a limitation in the interpretation of NMR results; however, without DMSO-*d*_6_, it was inevitable to minimize the D_2_O exchange reactions and monitor all of the proton signals.

**Figure 3:**
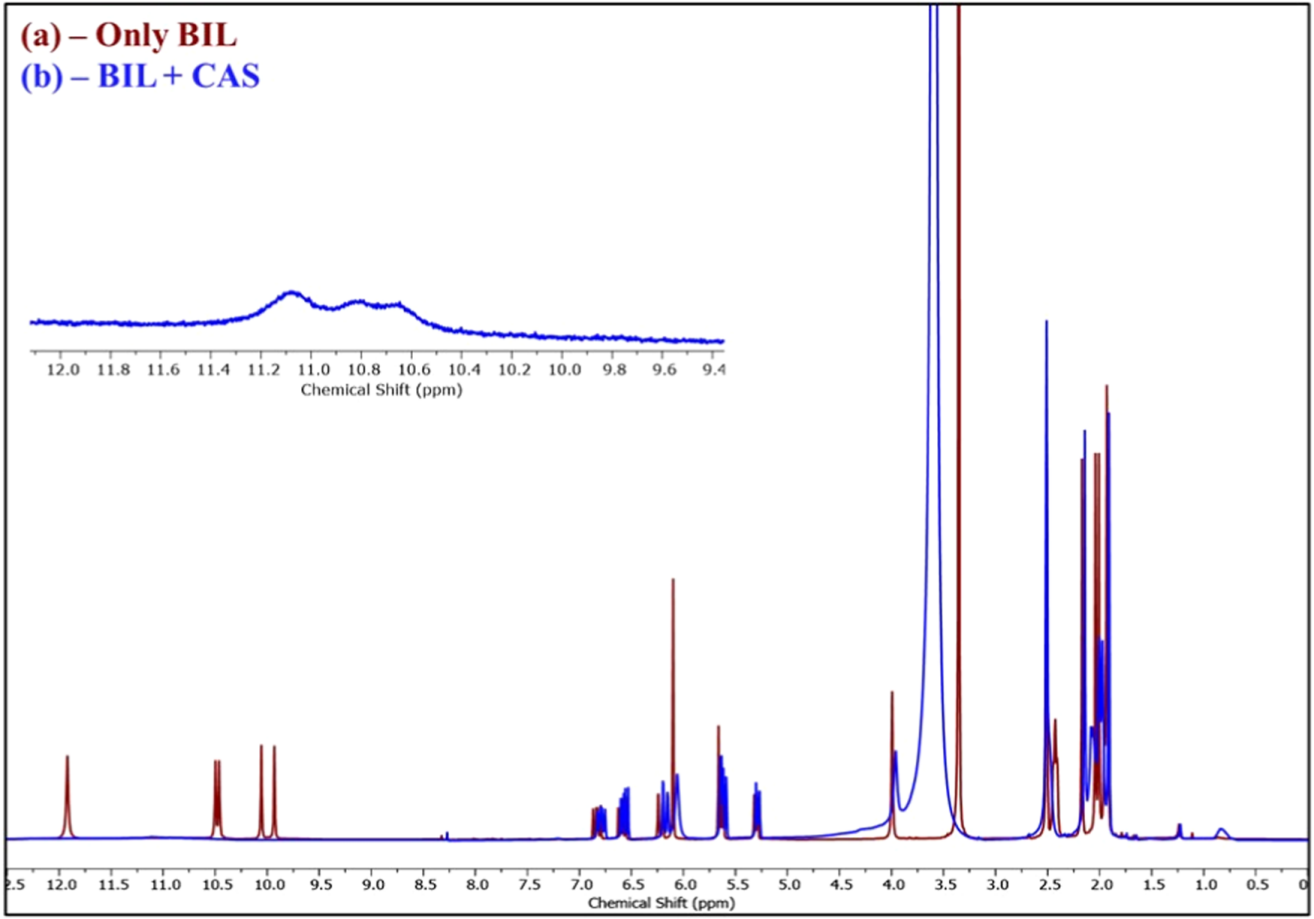
^1^H NMR spectrum (400 MHz) of BIL (10 mg/mL) in the (a) absence and (b) presence of CAS (1 mg/mL) in DMSO-*d*_6_.

### 2.9 CMC Point Modulation of Micelles

Pyrene exists as an essential hydrophobic molecule that strongly probes the formation of micelles.^34^ Because of its strong solvent dependence shown in its photophysics, we utilized it to detect the changes in the CMC of the CAS protein in the presence of BIL. The peak maxima in the excitation spectra of pyrene at λ_em_ = 390 nm are known to undergo a red shift from 332 to 335 nm when the molecule’s local environment changes from hydrophilic to lipophilic. The correlation of intensities at these wavelengths is then used to determine the CMC of micelle formation.^35,36^ Figure S14a illustrates the increment in the intensity of fluorescence excitation spectra recorded at 390 nm emission for pyrene in the presence of the CAS protein. On changing the polarity toward a hydrophobic environment, there is a clear red shift in the peak maxima from 332 to 335 nm (Figure S14b). On plotting I_335_/I_332_ with CAS concentration, we determined the induced changes in CMC in the presence of BIL. Figure S15 depicts the change in the CMC of CAS micelles when the BIL is introduced to them. For only CAS micelles, pyrene showed CMC at 0.84 mg/mL in PB at pH = 7.3. On adding BIL, a clear shift of CMC to a higher value was observed for CAS. For 15 μM BIL, CMC was found to be 1.28 mg/mL, and for 30 μM BIL, it was found to be 1.8 mg/mL. So, this confirms the effect of BIL interaction on the CAS structures in intrinsic fluorescence results, and it can be said that BIL induces structural changes in the CAS micelles, leading to changes in steady-state fluorescence and time-resolved fluorescence. To confirm and visualize the structural changes, we also performed AFM measurements.

### 2.10 Morphology Measurements by AFM

Force measurements have been an advantageous technique compared with other microscopies in terms of noise resistance and high-resolution analysis. One of the scanning-probe-based techniques is AFM, which is used in this study for determining the morphological changes in CAS micelles when BIL is introduced. Figure 4a,b shows the 2D and 3D morphological arrangements of CAS micelles (2 mg/mL) over the scan size of 20 μm × 20 μm (side of the square). It can be seen that the shape of CAS micelles is not fixed like that of spherical conventional micelles, such as that of sodium dodecyl sulfate (SDS). The average size and height can be recorded to be uniform near 100–300 nm, as reported in the earlier literature also.^37^ Figure 4c,d illustrates the changes in the morphology of CAS micelles in the presence of 100 μM BIL. There are two major changes observed when BIL is introduced. First, the bright yellow spots for blank CAS having a height of ∼150–200 nm (color bar) decreased to darker spots of 50–60 nm when BIL is introduced. The second observation is the modulation of the shape of the micelles, as can be seen from Figure 4d. The well-arranged pattern of CAS micelles in Figure 4b was completely diminished when BIL was introduced. So, the micelle distribution was greatly affected when BIL interacted with CAS micelles. These results support the decrease in the CMC of CAS micelles observed in an earlier section using pyrene. The intrinsic fluorescence of CAS micelles was greatly quenched when BIL was added to the micelles, which indicated that the BIL interaction with micelles was such that it tended to modulate and destroy/diminish the size of micelles. A deeper understanding can be given by the roughness parameters for these samples (Table 2). The surface roughness given by average roughness (*R*_a_) and root-mean-square roughness (*R*_q_) was found to be greatly affected. The values for CAS micelles were 40.9 and 51.1 nm, respectively, which decreased to 26.6 and 33.8 nm for BIL-modified micelles. So, these values confirm the decrease in the surface roughness due to micelles by BIL, thus inducing smoothness that decreases *R*_a_ and *R*_q_ values.

**Figure 4:**
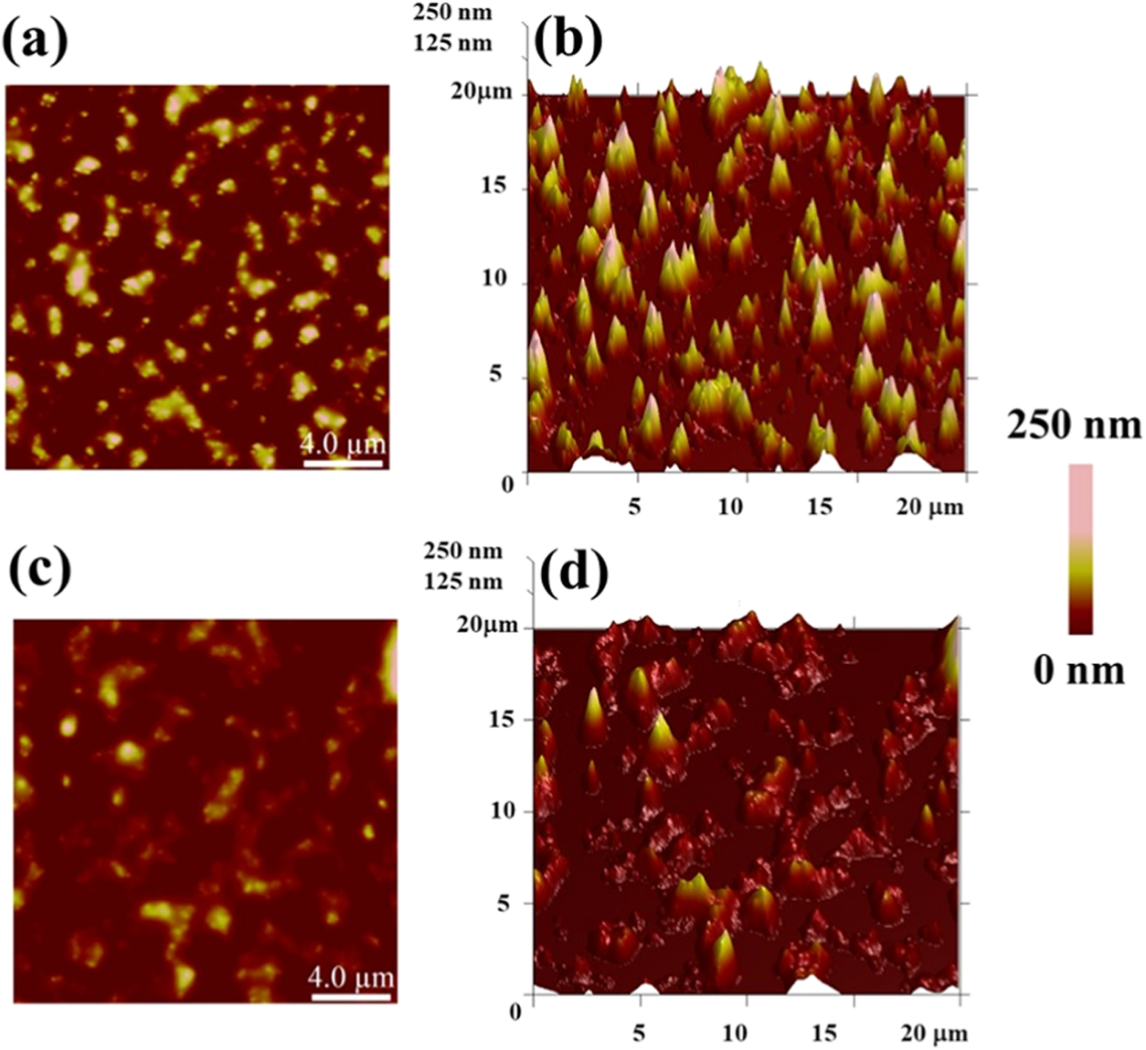
AFM images depicting 2D and 3D morphologies of (a, b) only CAS (2 mg/mL) and (c, d) CAS (2 mg/mL) with BIL (100 μM). Scale bar: 4 μm.

**Table 2:** *R*_a_ and *R*_q_ Distance Determined by AFM for CAS (2 mg/mL) and CAS (2 mg/mL) with BIL (100 μM)^a^

sample	*R*_a_ (nm)	*R*_q_
CAS 2 mg/mL	40.9	51.1
CAS 2 mg/mL +100 μM BIL	26.6	33.8

a *R*_a_ is the average surface roughness and *R*_q_ is the root-mean-square surface roughness.

### 2.11 Determination of Binding Location by an Extrinsic Probe

8-Anilinonaphthalene-1-sulfonic acid (ANS) is a weakly fluorescent probe due to its intramolecular charge transfer (ICT) process. Exceptionally, on interacting with a hydrophobic environment, such as protein pockets, this ICT process slows down to a great extent, which leads to a huge enhancement in the fluorescence with a blue shift from 520 to 460 nm. Any external molecule that has more capacity toward the ANS-bound pocket will dislodge ANS, which will easily be detected by fluorescence quenching.

Figure 5a depicts the fluorescence spectra of CAS-bound ANS with a gradual increment of BIL. For CAS, we have taken a higher concentration (2 mg/mL) than its CMC to ensure micelle formation. With this, when ANS is bound, the fluorescence of ANS drastically increases with a blue shift from ∼517 to 470 nm. Since the ICT process stops in ANS due to hydrophobicity in its local environment, the binding location of ANS will probably be the micellar hydrophobic cavity. To this system of CAS-confined ANS, we gradually started adding BIL. With even a small amount of BIL (1.1 μM), the fluorescence intensity at 470 nm (λ_ex_ = 350 nm) showed a huge decrease in its value. On further increasing the BIL concentration, it kept on decreasing (inset of Figure 5a). The decrease in intensity was also observed for the excitation spectra collected at λ_em_ = 470 nm (Figure S16). This clearly indicates that BIL displaces ANS from its binding site. This displacement decreases the number of CAS-confined ANS complexes in the solution reflected in the emission spectra. So, these changes confirm that the binding location of BIL in CAS is hydrophobic in nature, i.e., encapsulating pocket in the protein micelle. With the help of time-resolved measurements, this observation can further be supported.

**Figure 5:**
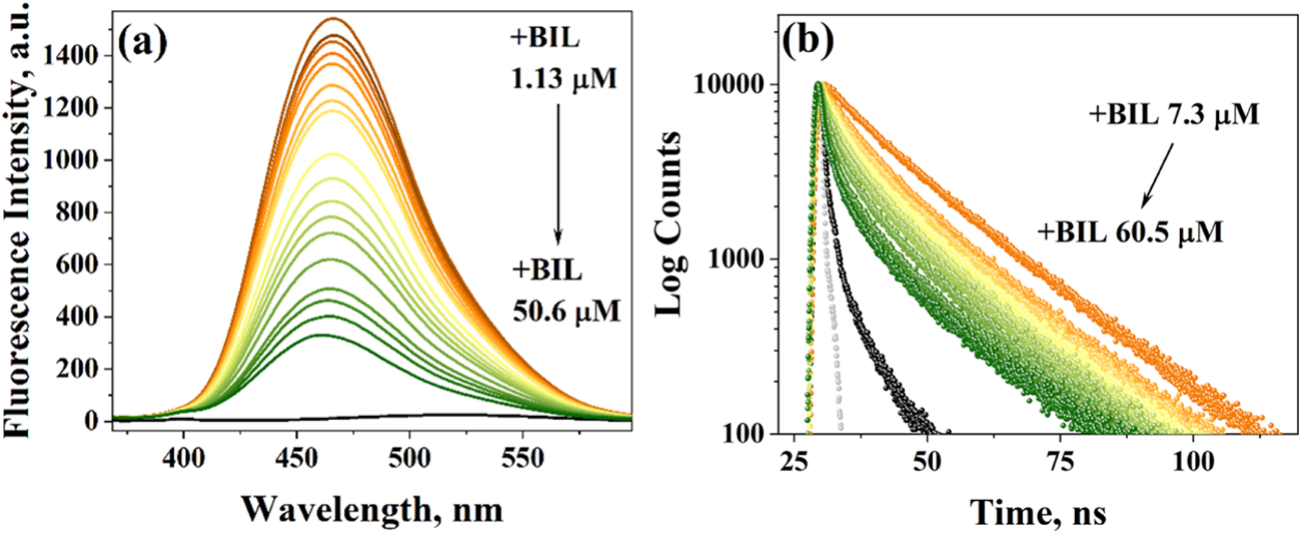
(a) Emission spectra (λ_ex_ = 350 nm) of the CAS (2 mg/mL)-bound ANS (26 μM) complex in the absence and presence of BIL (1.13–50.6 mM). The black-colored plot indicates that of only ANS (26 μM). (b) Fluorescence lifetime decay of the CAS (2 mg/mL)-bound ANS (26 μM) complex, in the absence and presence of BIL (7.3–60.5 μM) (λ_ex_ = 371 nm and λ_em_ = 470 nm). The black-colored plot indicates that of only ANS (26 μM), and the gray plot indicates that of IRF. Measurements are done in PB (pH = 7.3) at 298 K.

Figure 5b represents the time-resolved decay profiles of CAS-bound ANS in the absence and presence of BIL. Only ANS has a sharp decay with a fast lifetime component of 200 ps. Interestingly, when it binds to a protein, the fluorescence decay sharply slows down to behave as a triexponential decay.^38^ In the three lifetime components, the fast picosecond component is sourced from free ANS molecules that remained unbound in the solution. The other two components originate from the delay in the ICT process due to the protein binding. The lifetime component ranging from 2 to 7 ns is due to surface binding and is exposed to the hydrophilic solvent. The longest lifetime component of 10–17 ns is due to confinement of ANS in the hydrophobic pocket of the protein and is shielded from solvent exposure.

Table 3 lists the lifetime components of CAS-bound ANS derived from the decay profiles in Figure 5b. The fast picosecond component of 0.5–0.6 ns originated from free ANS molecules in the solution. The other two components of 7.7 and 18.9 ns can be due to binding with the protein surface and micelle pocket, respectively. The decay profiles in Figure 5b depict that the addition of BIL to CAS-confined ANS makes their excited-state complex decay at a faster pace. The average lifetime τ_av_ can also be seen to undergo a huge decrease of ∼ 8 ns in its value when the BIL is introduced. The changes in the pre-exponential factor of lifetime components in Table 3 suggest that the decrease in τ_av_ is mainly due to an increase in the α_1_ of the fast component and a decrease in the α_3_ of the longest component (Figure S17b). So, the hydrophilic lifetime component (free ANS) increases/is exchanged with the hydrophobic component (CAS-confined ANS) with the least effect on the second component amplitude (α_2_). So, the confined ANS is getting displaced by BIL, and the free ANS goes to the solution, leaving the BIL in the hydrophobic pocket of the protein micelle. ANS is a highly fluorescent probe only when it is bound to the hydrophobic protein pocket, which in this case is the micelle hydrophobic core. Due to the more binding affinity of BIL than that of ANS, it gets displaced from the core by BIL, which leads to a decrease in the fluorescence intensity and lifetime.

**Table 3:** Fluorescence Lifetime Decay of the ANS (26 μM) with CAS (2 mg/mL) Complex, in the Absence and Presence of BIL (7.3–60.5 μM; λ_ex_ = 371 nm and λ_em_ = 470 nm)^a^

sample	τ_1_ (ns)	α_1_	τ_2_ (ns)	α_2_	τ_3_ (ns)	α_3_	τ_av_ (ns)	χ^2^
ANS + 2 mg/mL CAS	0.51	0.39	7.67	0.2	18.86	0.41	9.4	1.05
+BIL 7.3 μM	0.65	0.48	6.43	0.25	18.1	0.27	6.753	1.09
+BIL 12 μM	0.65	0.53	6.15	0.24	17.9	0.23	5.9	1.2
+BIL 17.8 μM	0.63	0.58	5.63	0.23	17.51	0.19	5.05	1.17
+BIL 24.6 μM	0.66	0.63	5.42	0.21	17.4	0.16	4.32	1.13
+BIL 32.2 μM	0.66	0.68	5.26	0.19	17.3	0.13	3.72	1.19
+BIL 40.7 μM	0.66	0.74	5.03	0.16	17.17	0.1	3.06	1.06
+BIL 50.8 μM	0.61	0.8	4.54	0.13	17.06	0.07	2.34	1.1
+BIL 60.5 μM	0.56	0.84	4.3	0.1	17.1	0.05	1.84	1.1

a Measurements are done in PB (pH = 7.3) at 298 K.

### 2.12 Photophysical Study of Other Hydrophobic Ligands with CAS

The fluorescence enhancement of BIL in the presence of CAS was also compared with other ligands such as ANS, pyrene, 6-hydroxyflavanone (6-HF), 3-hydroxyflavone (3-HF), and sanguinarine (SANG). These molecules were chosen based on their well-known hydrophobic character, aromaticity, and hydrogen bonding capabilities. Figure S18 depicts the fluorescence spectra of these molecules in the presence of CAS. Like BIL, the fluorescence of all of the molecules was found to be enhanced upon adding the CAS protein. The increment patterns (Figure S19a) were similar to each other but were accompanied by differences in the shift of emission wavelength maxima. For example, ANS, 6-HF, and SANG underwent blue shifts (47, 4,0 and 20 nm, respectively), 3-HF underwent a red shift (24 nm), and pyrene underwent no visible shift when CAS was introduced to these molecules. Pyrene is a highly hydrophobic molecule with negligible water solubility due to no functional groups in its aromatic structure. On adding the CAS protein, its fluorescence increases, though with no shift. This clearly states that hydrophobic interaction leads to an increase in ligand fluorescence. SANG has hydrophobic (415 nm) as well as hydrophilic (565 nm) forms in the solution at this pH.^39^ On adding CAS, the peak at 415 nm underwent enhancement, whereas the peak at 565 nm underwent quenching (Figure S19b). This clearly states that the fluorescence enhancement observed with the other ligands, including BIL, is due to the hydrophobic interaction with the CAS protein. As stated, in the case of other molecules, along with the increase, there is a shift in their emission maxima. The blue shift can be attributed to the encapsulation of the ligand in a comparatively more hydrophobic environment, whereas the red shift is attributed to a lesser hydrophobic environment. The difference in the type of fluorescence signal is due to the difference in the electronic conjugation in their aromatic rings and the presence of different functional groups (sulfonic in ANS; hydroxy and carbonyl in 6-HF and 3-HF; quinoline ring in SANG). These functional groups differ from each other with different hydrogen bonding and solvent interaction capabilities that induce changes in the fluorescence profile of these ligands when the CAS protein is added. Similar to these, BIL is also a hydrophobic molecule, evident from its insolubility in water, but has carboxyl, carbonyl, and vinyl functional groups. So, the fluorescence enhancement observed in BIL involves a major hydrophobic interaction in combination with the interaction of these functional groups with the protein pocket of the CAS, and these findings were further well supported by the results of molecular docking given in Table S2. This study gives a more comprehensive understanding of how the CAS micelles encapsulate other ligands and will be useful for sensing and drug delivery applications.

### 2.13 MD Simulations

To interpret experimental observations, the interactions between β-casein (β-CAS) and the ligand BIL are critical. Understanding protein–ligand interactions at the molecular level presents challenges in experimental studies, which are often complemented by computational approaches. In this study, molecular dynamics (MD) simulations were employed to investigate the dynamics of β-CAS with BIL, focusing on molecular interactions that align with experimental findings. Since the crystal structure of β-CAS is not available in the Protein Data Bank (PDB), an initial model was generated using the RoseTTAFold server.^40^ The predicted structure displayed significant random coil and helical regions, indicative of an unfolded conformation. To enhance stability and identify a likely conformation, the model was simulated for a 1 μs MD simulation. Root-mean-square deviation (RMSD) analysis (Figure S20a) indicated minimal fluctuations, particularly in the final 200 ns. Using MDAnalysis,^41^ the average structure during the 0.8–1 μs time frame was extracted, validated for structural integrity, and subsequently docked with BIL using AutoDock Vina.^42^ Docking revealed three binding hotspots (Figure 6a,b), and docked complexes were further analyzed through 1 μs MD simulations for their stability. The RMSD plots (Figure S20b) showed that complexes 1 and 2 maintained RMSD values below 2 nm, whereas complex 3 exhibited instability with BIL dissociating after 250 ns. Noncovalent interaction snapshots (Figures S21 and S23) highlighted a predominantly hydrophobic environment (green boundary) stabilizing complexes 1 and 2, whereas complex 3 lacked this uniform environment, leading to ligand dissociation from the protein. Hydrogen bonding analysis further supported these findings. Complex 2 consistently maintained H-bonds with hydrophobic amino acids, such as Met-171, Phe-172, and Pro-173 (Figure 6c), whereas complex 1 showed transient interactions. The four lactam nitrogen atoms of the BIL were found to be in the hydrogen bonding range (Figure 6b). Detailed H-bond analysis revealed stable bonds for Met-171 and Pro-173, with distances below 2 Å, while H-bonds with Phe-172 were less stable, as shown in Figure S24. The mean number of H-bonds across simulations was highest for complex 2 (2.84), followed by complex 1 (2.3) and complex 3 (0.8), making hotspot 2 the most probable binding site. Additionally, in complex 2, Trp-158 was found to exhibit noncovalent interactions, namely, hydrophobic and van der Waals, with BIL, and Figure S25 depicts their binding modes responsible for FRET in experiments. The probability distribution function of these interactions gave the energy profiles of the short- and long-range interactions involved in complex 2 (Figure S26). So, the hydrophobic interactions found from fluorescence experiments and hydrogen bonding from NMR and UV–vis measurements are further corroborated and characterized by simulation studies.

**Figure 6:**
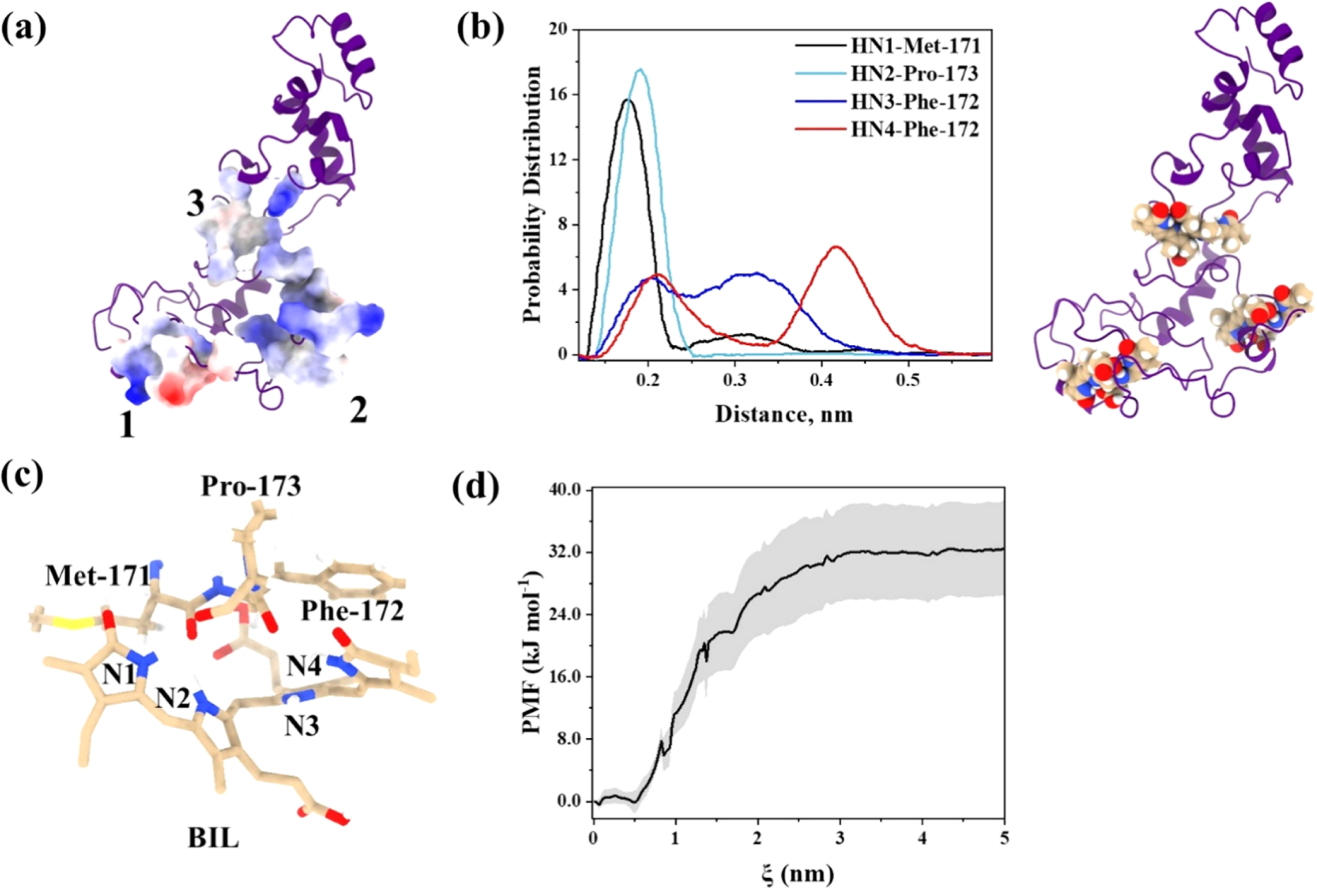
(a) Electrostatic potential for the three locations processed for MD simulations. (b) Binding hotspots of BIL in the protein and probability distribution plot of the distance between ligand nitrogen atoms and interacting amino acids at location 2. (c) H-bonded BIL with amino acids in location 2. (d) PMF curve for the dissociation of BIL from CAS in complex 2. The shaded region indicates the standard error calculated from five average simulations.

Umbrella sampling simulations were performed to estimate the binding free energy and validate the stability of complex 2. Steered molecular dynamics (SMD) determined the peak pulling force required to dissociate BIL (554.982 kJ mol^–1^ nm^–1^, Figure S28). Potential of mean force (PMF) curves (Figure 6d) were calculated using the weighted histogram analysis method (WHAM) and were averaged over five independent simulations. This yielded a binding free energy of −32.9 kJ/mol, in agreement with experimental values (−24.56 kJ/mol) after accounting for contributions from other casein proteins (α-CAS and κ-CAS). These results substantiate the higher binding affinity of BIL for β-CAS.

The hydrophobic amino acid environment significantly influenced BIL’s photophysical properties, which were investigated through time-dependent density functional theory (TDDFT) calculations. Snapshots of protein–ligand complexes from the MD trajectory were extracted at 100 ns intervals, and excited-state calculations were performed. The computed absorption spectra for BIL (437 nm) closely matched the experimental values (438 nm). Protein environments induced a red shift in the time average absorption spectra (complex 2:461 nm, complex 1:457 nm, complex 3:444 nm); since complex 3 does not interact with any amino acids, it is closer to the independent BIL, which is 437 nm, confirming the role of amino acid interactions in altering electronic properties. HOMO–LUMO diagrams (Figure 7) further illustrate changes in orbital shapes due to the protein environment. These results corroborate experimental findings and highlight the synergy of MD and TDDFT in advancing our understanding of protein–ligand interactions, providing novel insights into the β-CAS–BIL system.

**Figure 7:**
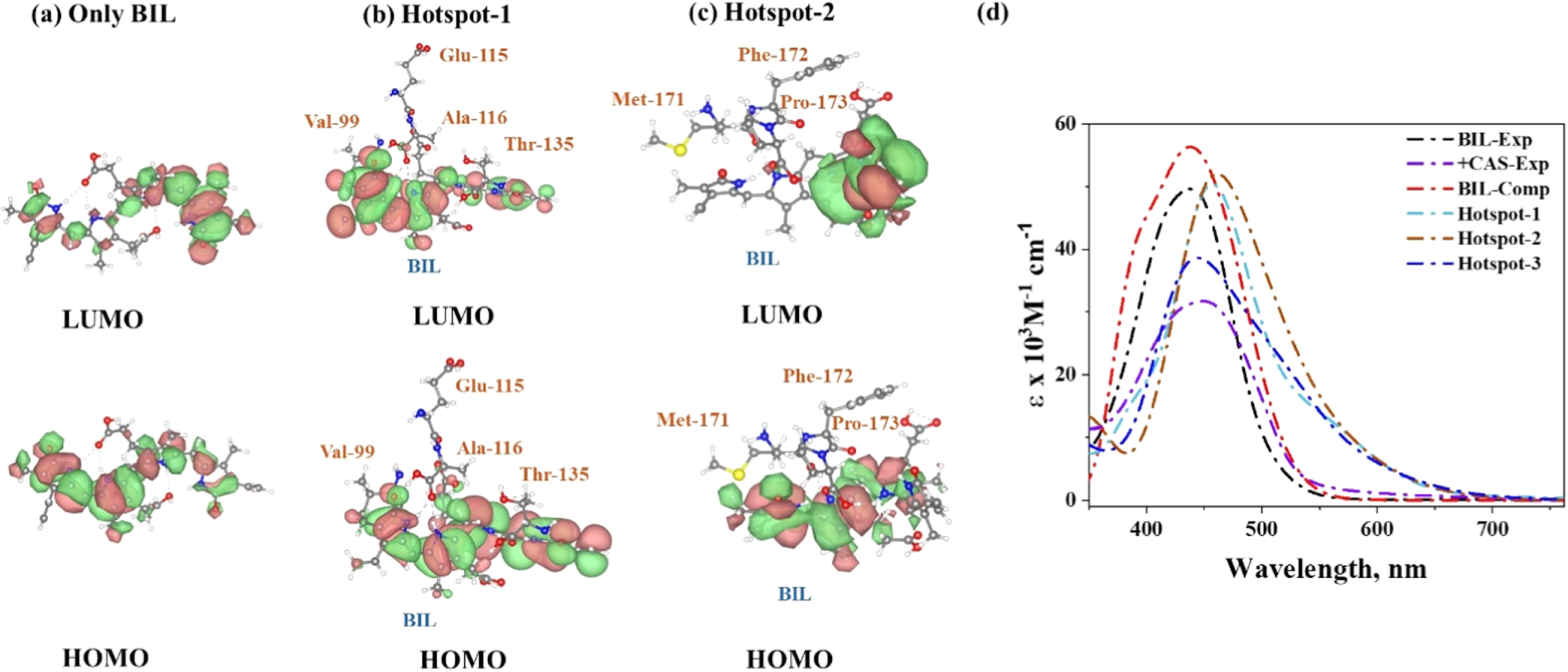
Orbital diagram picture of (a) only BIL without an amino acid environment and (b) BIL-amino acids in hotspot 1 and (c) hotspot 2. (d) Comparison between experimental and computed absorption spectra.

## 3 Conclusions

Using spectroscopic techniques, we characterized the interaction of BIL with the CAS protein. The red shift and decrease in absorption of BIL get saturated when CAS micellization takes place. This confirms the encapsulation of BIL inside CAS micellar pockets. The fluorescence of BIL underwent a huge increase inside these micellar pockets in comparison to that without micellization. Using CD spectra, the changes in the conformation of BIL were determined when CAS CMC was achieved. The conformational change was determined to be responsible for the changes in BIL photophysics. Intrinsic fluorescence of CAS was found to be greatly affected when BIL was introduced. It indicates changes in the CAS micellar structure. The FRET mechanism was determined to be a contributing factor to the photophysical changes. The specific interactions of BIL, when CAS encapsulated it, were determined by the ^1^H NMR study. Changes in the pyrene emission characterized the shift of the CMC of the CAS protein and indicated structural changes in CAS micelles. AFM measurements determined the decrease in the surface roughness due to disruption in CAS micellar shapes when BIL was introduced. Replacement of ANS from the micellar pocket confirms the encapsulating location of BIL inside CAS micelles. The hydrophobic forces of CAS micelles responsible for the fluorescence enhancement in BIL were also investigated by comparing the photophysics with the interaction of CAS and ANS, pyrene, 6-HF, 3-HF, and SANG molecules. The MD simulation study gave a detailed analysis of the molecular interactions between BIL and the β-CAS protein. Electronic properties were determined for BIL in the three hotspots using TDDFT calculations. The present study will provide a detailed understanding of the influence of milk proteins, like casein, in future medicinal therapies, since BIL content produced by liver functioning can be affected by milk proteins.

## Supplementary Material



## Notes

Experimental section; fluorescence spectra; synchronous fluorescence spectra; fluorescence lifetime decay; FRET donor–acceptor overlap; pyrene fluorescence; and MD simulation analysis (PDF)

## Abbreviations

BIL: bilirubin
CAS: casein
CMC: critical micelle concentration
Trp: tryptophan
Tyr: tyrosine
FRET: Förster resonance energy transfer
ANS: 8-anilino-1-naphthalenesulfonic acid
NMR: nuclear magnetic resonance
AFM: atomic force microscopy
