# Supplementary material for: Elucidation of the Synergistic Interaction Between Bilirubin and Casein Protein: An Integrated Spectroscopy and Computational Approach
Source: Biomacromolecules. 2025 Jul 23;26(8):5298–309. doi: 10.1021/acs.biomac.5c00795 (PMC12344696; doi:10.1021/acs.biomac.5c00795)
Supplement: Supplementary file 1 [file bm5c00795_si_001.pdf]

# Supplementary Information

# Elucidation of Synergistic Interaction Between Bilirubin and Casein Protein: An Integrated Spectroscopy and Computational Approach

Sudhanshu Sharma[a], Jyoti Vishwakarma[a], Jacek Czub[c,d], Subrahmanyam Sappati[b,d*], Krishna Gavvala*[a]

[a]Department of Chemistry, Indian Institute of Technology Hyderabad, Kandi, Sangareddy, Telangana-502284, India.

[b]Department of Pharmaceutical Technology and Biochemistry, Ul. Narutowicza Str. 11/1280-233 Gdańsk Poland

[c]Department of Physical chemistry, Ul. Narutowicza Str. 11/1280-233 Gdańsk Poland

[d]BioTechMed Center, Ul. Narutowicza Str. 11/1280-233 Gdańsk Poland

* Corresponding authors. E-mail: kgavvala@chy.iith.ac.in and subsappa@pg.edu.pl

# MATERIALS AND METHODS

## Materials

CAS protein (CAS no. 9000-71-9) and BIL (CAS no. 635-65-4) were obtained from Sisco Research Laboratories Pvt. Ltd. (SRL) – India and Sigma Aldrich, respectively. Pyrene (CAS no. 129-00-0) was obtained from Alfa Aesar. Sodium 8-Anilino-1-naphthalenesulfonate (ANS) salt (CAS no. 1445-19-8) was obtained from TCI Chemicals. 6-hydroxyflavanone (6-HF) (CAS no. 4250-77-5) was purchased from Sigma Aldrich, 3-hydroxyflavone (3-HF) (CAS no. 577-85-5) was purchased from TCI Chemicals, sanguinarine (SANG) chloride hydrate (CAS no. 5578-73-4) was purchased from TCI Chemicals, sodium phosphate monobasic (CAS no. 7558-80- 7) and dibasic (CAS no. 7558-79-4) anhydrous were obtained from Srichem. For all measurements and stock solutions, phosphate buffer (PB) (0.1 M, pH = 7.3) was used and temperature was kept at 298.15 K unless otherwise mentioned. Since BIL degrades easily within 2-3 days, for every measurement, freshly prepared solution of BIL was made in 0.16 M NaOH and then diluted in 0.1 M PB to prepare desired stock solution at pH = 7.3.

## Methods

### Steady-state absorption and fluorescence spectroscopy

UV-Vis absorption measurements were carried out using JASCO-V730 spectrophotometer. The concentration of BIL was estimated by using molar extinction coefficient of BIL, $\varepsilon_{440\ nm}$ = 47, 500 $M^{-1}$ $cm^{-1}$ at 440 nm.[1] Fluorescence spectra were measured using JASCO-FP8350 spectrofluorometer. BIL fluorescence spectra was analyzed by exciting BIL at $\lambda_{ex}$ = 440 nm. The slit width was maintained at 5/10 nm and scan range was kept at 455-740 nm for the measurement of BIL in presence of CAS. Intrinsic fluorescence of CAS micelles was measured using excitation wavelength of $\lambda_{ex}$ = 280 nm with slit width of 2.5/5 nm at scan range of 295-540 nm.

Synchronous fluorescence spectra were collected by fixing the Stoke's shift of amino acid, at $\Delta\lambda$ = 60 nm for Trp with collection range 300-400 nm and at $\Delta\lambda$ = 15 nm for Tyr with collection range of 255-400 nm.

The concentration of ANS probe was calculated using molar extinction coefficient, $\varepsilon_{350\ nm}$ = 4,950 $M^{-1}$ $cm^{-1}$ in absorption spectra at 350 nm. For monitoring ANS fluorescence bound to CAS micelles, the excitation wavelength was $\lambda_{ex}$ = 350 nm with slit width maintained at 5/10 nm and scan range was kept at 365-685 nm. For monitoring changes in CMC of CAS, the

excitation spectra of extrinsic probe, pyrene (1.1 μM) was used. The slit width was maintained at 2.5/5 nm and excitation spectra was collected at $\lambda_{em}$ = 390 nm with scan range of 200-375 nm.

Inner filter effect was corrected in all the fluorescence quenching results, using equation[2],

$$F_{corr} = F_{obs} \times \exp(A_{ex} + A_{em})/2 \quad (1)$$

where $F_{corr}$ is corrected fluorescence intensity and $F_{obs}$ is observed fluorescence intensity. $A_{ex}$ denotes the absorbance value at excitation wavelength and $A_{em}$ is the absorbance value at the emission wavelength.

Hill1 equation[3] for binding using absorbance,

$$A = A_0 + (A_{end} - A_0)\left(\frac{[CAS]^n}{k_D^n + [CAS]^n}\right) \quad (2)$$

where $A_0$ and $A$ denote absorbance of BIL at 500 nm in absence and presence of CAS and $A_{end}$ denotes absorbance saturated at end concentration of CAS. $k_D$ is the dissociation constant of the complex and n denotes the number of binding sites.

Hill1 equation for binding using fluorescence intensity,

$$F = F_0 + (F_{end} - F_0)\left(\frac{[CAS]^n}{k_D^n + [CAS]^n}\right) \quad (3)$$

where $F_0$ and $F$ denote fluorescence intensity of BIL at 525 nm in absence and presence of CAS and $F_{end}$ denotes fluorescence intensity saturated at end concentration of CAS. $k_D$ is the dissociation constant of the complex and $n$ denotes the number of binding sites.

**Circular dichroism spectroscopy**

To monitor the changes in chiral behavior of BIL, we utilized Jasco-J815 spectrophotometer for measuring CD spectral changes in presence of CAS at 298.15 K. The scan range was maintained at 300-650 nm where only BIL absorbs without interference of protein and the scan speed was kept at 200 nm/min with two accumulations of spectra.

**Thermodynamics parameters measurements**

Using temperature-dependent fluorescence measurements, the thermodynamic parameters were derived.

Stern-Volmer equation,[4]

$$\frac{F_0}{F} = 1 + K_{sv}[BIL] = 1 + k_q\tau_0\,[BIL] \;\; or \;\; \frac{F_0 - F}{F} = K_{sv}[BIL] = k_q\tau_0\,[BIL] \tag{4}$$

where $F_0$ and $F$ correspond to the fluorescence intensity of CAS in the absence and presence of BIL respectively, $K_{sv}$ denotes the Stern-Volmer quenching constant, depending on the type of interaction, it can be static or dynamic, $k_q$ denotes the apparent bimolecular quenching constant and $\tau_0$ corresponds to the average lifetime of CAS i.e. 3.57 ns.

Log form of Hill equation,[5]

$$Log\left(\frac{F_0 - F}{F}\right) = Log\,K_a + n\,Log\,[BIL] \tag{5}$$

where $K_a$ and $n$ are apparent binding constants and the number of binding sites, respectively.

Van't Hoff equation,

$$\ln K_a = -\frac{\Delta H}{RT} + \frac{\Delta S}{R} \tag{6}$$

where $K_a$ represents the binding constant at different temperatures. $R$ denotes the universal gas constant (8.314 J $mol^{-1}$ $K^{-1}$), and $T$ is the temperature in Kelvin.

Gibb's free energy equation,

$$\Delta G = \Delta H - T\Delta S \tag{7}$$

**Time resolved fluorescence spectroscopy**

Time resolved fluorescence lifetime measurements were recorded by a time-correlated single photon counting (TCSPC) technique from Horiba (Fluorohub) setup. The CAS sample was excited by using 291 nm NanoLED diode and ANS was excited by using 371 nm NanoLED using <1 ns pulsed (1 MHz) with 1.495 ns full-width half maxima (FWHM). The standard fit of any decay was assessed by keeping the minimum distribution of residuals and $\chi^2$ near 1. In TCSPC measurements, the mean error was less than or equal to 5%. The Instrumental Response Function (IRF), which was subsequently fitted to experimental decays was convoluted using the following exponential decay functions. All the obtained decays are fitted using DAS6 analysis software. The following equation were utilized to fit the time resolved fluorescence data,[4]

$$I(t) = \sum \alpha_i e^{\left(-\frac{t}{\tau_i}\right)} \tag{8}$$

where $I(t)$, $\alpha_i$ and $\tau_i$ are correspond to the fluorescence decay at time $t$, amplitude of $i^{th}$ components and fluorescence decay of $i^{th}$ components, respectively.

Dynamic quenching constant equation,[6]

$$\frac{\tau_0}{\tau} = 1 + K_D[BIL] \qquad (9)$$

where $\tau_0$ and $\tau$ denotes average lifetime of CAS micelles in absence and presence of [BIL]. $K_D$ denotes the dynamic quenching constant for the binding.

**Förster resonance energy transfer (FRET) measurements**

FRET was employed to study the energy transfer efficiency between the donor and acceptor molecule; here, CAS act as a donor and BIL act as an acceptor. The distance (*r*) between the donor CAS and acceptor BIL was calculated on the basis of Förster theory equation,

$$E = 1 - \frac{F}{F_0} = \frac{R_0^6}{R_0^6 + r^6} \qquad (10)$$

where, $E$ is described as energy transfer efficiency between the donor and acceptor, and $R_0$ is known as the Förster critical distance when the excitation energy transfer efficiency is 50% and could be estimated by equation,

$$R_0 = 0.211\left(\kappa^2 \times n^{-4} \times \phi \times J(\lambda)\right)^{1/6} \text{Å} \qquad (11)$$

Here, $\kappa^2$ is the spatial dipole orientation factor in the solution and is considered to be equal to 2/3, $n$ is the average refractive index of the medium (here, in water, n =1.33), $\phi$ is the fluorescence quantum yield of the donor ($\phi$ of CAS = 1.49)[7] and $J(\lambda)$ corresponds to the spectral overlap integral between donor emission and acceptor absorbance, and could be determined by equation,

$$J(\lambda) = \frac{\Sigma F(\lambda)\varepsilon(\lambda)\lambda^4 d\lambda}{\Sigma F(\lambda) d\lambda} \qquad (12)$$

where, $F(\lambda)$ represents the corrected fluorescence intensity of the donor at wavelength $\lambda$ and $\varepsilon(\lambda)$ is the molar absorption coefficient of the acceptor at wavelength $\lambda$.

**NMR spectroscopy**

$^1$H NMR measurements were performed using AVANCE III 400 FT-NMR (Bruker, Switzerland) Spectrometer operating at a frequency of 400 MHz and equipped with an RT-probe. Deuterated DMSO-$d_6$ was utilized as the solvent for all the NMR studies. The BIL samples were taken with a concentration of 10 mg/ml in absence and presence of CAS (1 mg/ml). NMR spectrum of CAS (1 mg/ml) without BIL was also measured as a control

spectrum. The measurements were performed by using ~0.6 ml of the samples in 5 mm NMR tubes at 297.4 K. The acquired data was analysed by using MestReNova software[8].

**Atomic force microscopy**

The morphology of CAS micelles with and without BIL were analyzed using an AFM instrument (Multimode 8 equipped with NanoScope V controller). For imaging, TAP525A phosphorus (n) doped silicon cantilever (force constant = 200 N/m) was used, and the morphology and roughness parameters were processed by NanoScope Analysis 3.00 software. The concentration of CAS was 2 mg/ml and BIL was 100 μM. For AFM analysis, the 10-20 μL of CAS alone and CAS with BIL were drop-casted onto glass slides and left overnight for drying under vacuum conditions. Following that, non-contact tapping mode AFM analysis was performed on the prepared samples.

**MD simulation**

2D structure of BIL molecule was drawn in ChemDraw Ultra 12.0 and then converted into mol2 3D structure by Chem3D Pro 12.0 where geometry was optimized by energy minimization with minimum RMS gradient of 0.01 using MM2 method. The final structure then subjected to Gaussian 16 software package to obtain the probable geometry present at the global minima in ground state of BIL. In the structure optimization, B3LYP functional and basis set of 6-311+g(d,p) was used.[9]

MD simulation was performed to obtain the most probable structure of β-CAS protein. Since crystal structure of β-CAS is not available on protein data bank (PDB), the initial structure was predicted by using RoseTTAFold server by providing the amino acid chain sequence.[10] Then the structure obtained was used in Gromacs package[11] with the help of CHARMM36m force field[12] for minimizing and simulating β-CAS protein in water for a time period of 1 μs. TIP3P water model was used to solvate the protein. $Na^+$ and $Cl^-$ ions were used to neutralize the protein charges and introducing 0.16 M salt concentration in a cubic box. The steepest descent approach was used to minimize the system's energy by 50,000 steps until the highest force, $F_{max}$ was less than 1000 $kJmol^{-1}$. Particle Mesh Ewald (PME)[13] approach was applied for long-range interactions (grid spacing of 0.16 nm). Canonical ensemble with modified Berendsen thermostat[14] with velocity rescaling at 300 K and isothermal isobaric ensemble with Berendsen pressure coupling[14] at 1 bar were utilized to equilibrate NVT and NPT each for 400 ps, respectively. Cut-off ratios of 1.2 nm were used for coulomb and Van der Waals' potentials for

the calculation of short-range nonbonded interactions. With a time step of 2 fs, the MD run was performed for a period of 1 μs.

The average structure of 800-1000 ns of the protein was obtained using MDanalysis software package[15] and used it to perform MD run with BIL ligand. Using AutoDock Vina 1.2[16], BIL was docked to the average structure and gave three probable binding hostspots (**Fig. 9**). The simulation procedure is similar to above mentioned for only protein and three protein-BIL simulations were performed for a 1 μs time period using topology file obtained by SwissParam.[17, 18] Schrödinger Maestro 4.1 software[19] was used to visualize the protein amino acid environment around BIL.

**Umbrella sampling simulations**

In this study, steered molecular dynamics (SMD) simulations were applied to investigate the unbinding process of the BIL from the CAS binding site. These simulations were performed using the GROMACS package, where an external pulling force was applied along the z-axis to pull the BIL out of the binding site. A spring constant of 1000 kJ/mol/nm² and a pull rate of 0.01 nm/ps were applied over a 500 ps simulation period. The resulting SMD trajectories were analysed using 34 sampling windows, with distances ranging from 0.14 nm to 5 nm. To calculate the potential of mean force (PMF)[20], the umbrella sampling method was used. Each sampling window was equilibrated for 100 ps before being utilized as the starting configuration for umbrella sampling simulations. These simulations were conducted for 10 ns per window, resulting in a total simulation time of 280 ns across the 28 windows. To obtain unbiased data, all the trajectories were processed using the weighted histogram analysis method (WHAM)[21] which allowed for the calculation of the free energy profile along the reaction coordinate.

**Excited state calculations with Time-Dependent Density Functional Theory (TDDFT)**

To understand the spectroscopic aspects, Gaussian 16 software[9] was used to perform quantum chemical calculations for BIL with and without amino acid environment. We performed optimisation of BIL in the ground state with DFT calculations and then computed absorption spectra by TDDFT calculations.[22] The ground-state optimization and excited-state calculations were conducted using the B3LYP exchange correlation functional paired with the 6-311+g(d,p) basis set.[23] To reduce environmental effects, all calculations were performed in an adiabatic framework, employing water as the solvent modelled through the Integral Equation Formalism Polarizable Continuum Model (IEFPCM).[24] Theoretical UV-visible absorption spectra were

then generated based on TDDFT excited-state calculations. The same level of calculations was performed on BIL geometry extracted from MD simulation trajectories at 100 ns intervals from 0 to 700 ns.

For the three CAS-BIL complexes, three BIL were extracted along with the surrounding amino acid environment of the protein. BIL in complex-2 was extracted with only hydrogen bonded amino acids (Met-171, Phe-172 and Pro-173) whereas in complex-1 and complex-3, BIL was extracted with amino acids that are present in the vicinity radius of 2.5 Å. These three configurations then subjected to excited state calculations using TDDFT.

**Table S1.** Fluorescence lifetime decay of CAS (2 mg/ml) in presence of BIL (1.1-155.3 μM) ($\lambda_{ex}$ = 291 nm and $\lambda_{em}$ = 335 nm). Measurements are done in PB (pH = 7.3) at 298 K.

| **Sample** | $\tau_1$ **(ns)** | $\alpha_1$ | $\tau_2$ **(ns)** | $\alpha_2$ | $\tau_{av}$ **(ns)** | $\chi^2$ |
|---|---|---|---|---|---|---|
| CAS 2 mg/ml | 2.29 | 0.64 | 5.81 | 0.36 | 3.57 | 1.1 |
| +Bil 1.1 μM | 2.37 | 0.66 | 5.89 | 0.34 | 3.56 | 1 |
| +Bil 4.3 μM | 2.31 | 0.64 | 5.75 | 0.36 | 3.55 | 1.1 |
| +Bil 10.3 μM | 2.28 | 0.65 | 5.74 | 0.35 | 3.50 | 1.2 |
| +Bil 15 μM | 2.32 | 0.66 | 5.78 | 0.34 | 3.49 | 1.1 |
| +Bil 20.8 μM | 2.22 | 0.66 | 5.65 | 0.34 | 3.40 | 1.1 |
| +Bil 25.7 μM | 2.28 | 0.67 | 5.73 | 0.33 | 3.40 | 1.1 |
| +Bil 30.4 μM | 2.21 | 0.67 | 5.65 | 0.33 | 3.35 | 1.1 |
| +Bil 35 μM | 2.25 | 0.69 | 5.73 | 0.31 | 3.34 | 1.1 |
| +Bil 40.2 μM | 2.33 | 0.70 | 5.78 | 0.30 | 3.36 | 1.1 |
| +Bil 52.4 μM | 2.16 | 0.73 | 5.55 | 0.27 | 3.07 | 1.2 |
| +Bil 60.6 μM | 2.05 | 0.73 | 5.41 | 0.27 | 2.95 | 1.2 |
| +Bil 72.7 μM | 1.91 | 0.75 | 5.22 | 0.25 | 2.74 | 1.2 |
| +Bil 80.7μM | 1.85 | 0.76 | 5.13 | 0.24 | 2.65 | 1.3 |
| +Bil 92.7 μM | 1.78 | 0.77 | 5.05 | 0.23 | 2.55 | 1.1 |
| +Bil 100.6 μM | 1.87 | 0.78 | 5.18 | 0.22 | 2.60 | 1.2 |
| +Bil 120.4 μM | 1.76 | 0.79 | 5.07 | 0.21 | 2.46 | 1.3 |
| +Bil 136 μM | 1.76 | 0.80 | 5.08 | 0.20 | 2.44 | 1.3 |
| +Bil 155.3 μM | 1.67 | 0.80 | 4.96 | 0.20 | 2.34 | 1.3 |

**Table S2.** Molecular Docking Analysis of B-CAS protein docked with BIL ligand.

| Ligand | Binding Mechanism | Interaction Depth | Avg. Binding Energy | Absorption/Fluorescence | Remarks |
|---|---|---|---|---|---|
| **Bilirubin (BIL)** | Hydrophobic + H-bonds (COOH, NH, vinyl) | At Hotspot-2 in CAS | –32.9 kJ/mol *(from PMF); Hotspot 2* | Strong fluorescence enhancement; Redshift (to 461 nm in TDDFT; ~438 nm expt.) | Most stable, deeply embedded; validated via MD/TDDFT |
| **ANS** | Hydrophobic + weak H-bond (sulfonic group) | Surface or shallow cavities | –26 kJ/mol; Hotspot 2 | Fluorescence enhancement; Blueshift (~47 nm) | Easily displaced; commonly used FRET/micellar probe |
| **Pyrene** | Pure hydrophobic interaction | Deep (core) | -27 kJ/mol *(from hydrophobic packing); Hotspot 2* | Fluorescence enhancement; No shift | Hydrophobic probe; strong partitioning into micelle core |
| **6-Hydroxyflavanone (6-HF)** | Mixed H-bonding and hydrophobic | Mid-depth or semi-encapsulated | –27.7 kJ/mol *(inferred from shift and interaction flexibility); Hotspot 2* | Fluorescence enhancement; Blueshift (~40 nm) | Shows moderate encapsulation and conformational adaptation |
| **3-Hydroxyflavone (3-HF)** | Specific H-bond + keto–enol tautomer sensitivity | Mid-depth to site-specific | –26.5 kJ/mol *(estimated; more rigid binding); Hotspot 2* | Fluorescence enhancement; Redshift (~24 nm) | Stronger binding than 6-HF; tautomerism affects localization |
| **Sanguinarine (SANG)** | Electrostatic + π–π stacking (form-dependent) | Dual-form (environmentally responsive) | –30.4 and –30.9 kJ/mol *(iminium > alkanolamine); Between Hotspot1 &2* | Fluorescence enhancement (415 nm), Quenching (565 nm), Blueshift (20 and 7 nm, respectively) | Selective stabilization of hydrophobic alkanolamine form |

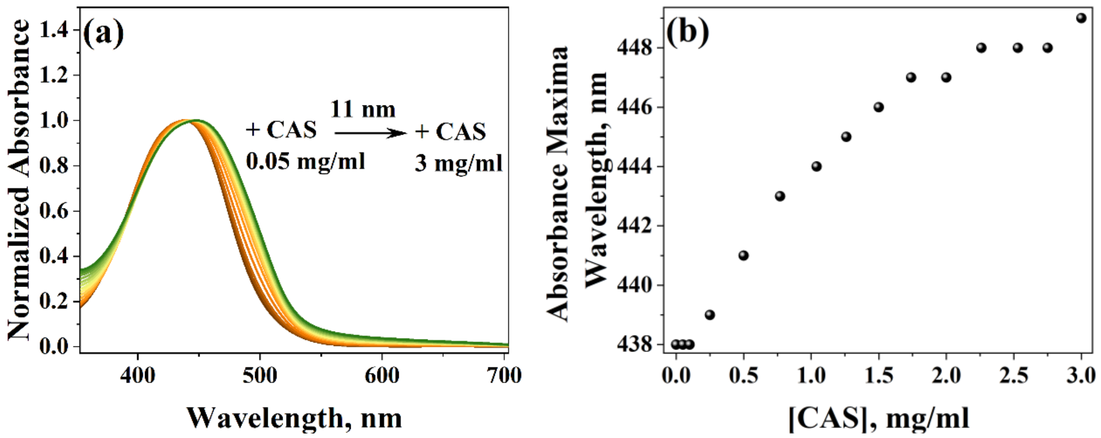


**Fig. S1.** (a) Normalized absorbance spectra of BIL in absence and presence of CAS. (b) Absorbance maxima wavelength of BIL as a function of CAS protein. Measurements are done in 0.1 M PB (pH = 7.3) at 298 K.

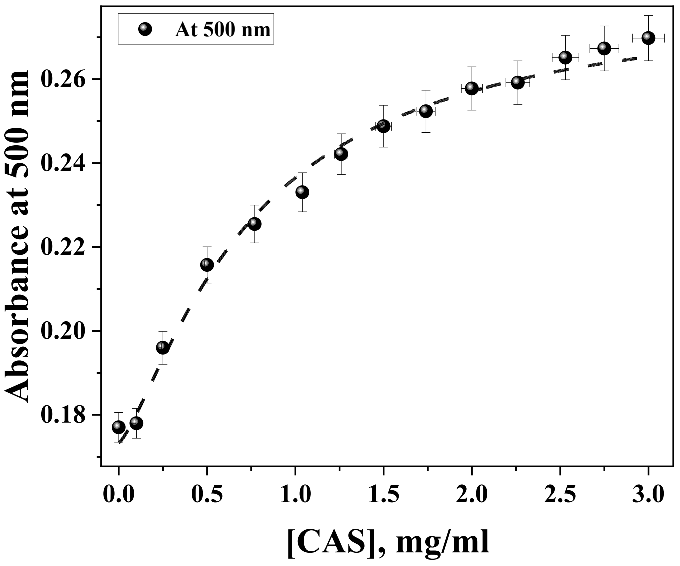


**Fig. S2.** Absorbance at 500 nm of BIL as a function of [CAS] protein. Measurements are done in 0.1 M PB (pH = 7.3) at 298 K.

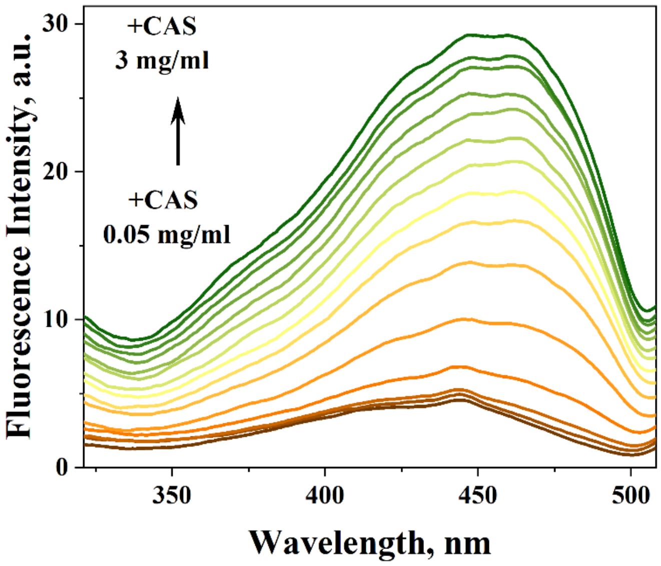


**Fig. S3.** Excitation spectra ($\lambda_{em}$ = 525 nm) of BIL (16.8 μM) in absence and presence of CAS (0.05 mg/ml to 3 mg/ml). Measurements are done in 0.1 M PB (pH = 7.3) at 298 K.

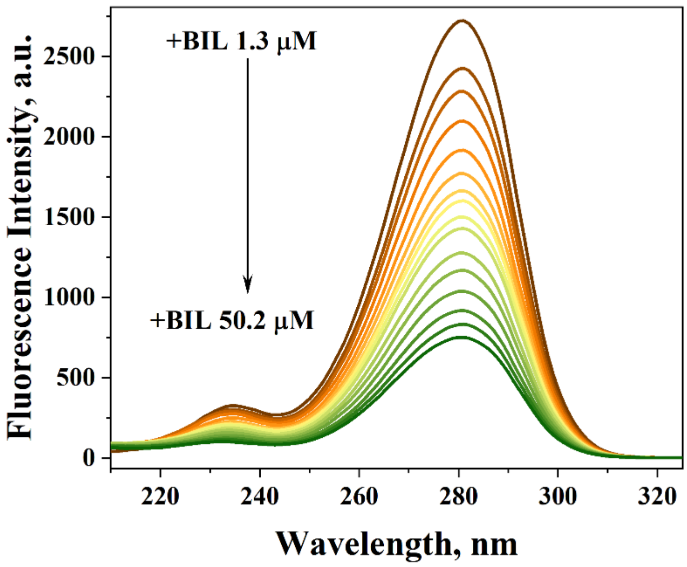


**Fig. S4.** Excitation spectra ($\lambda_{em}$ = 335 nm) of CAS protein (2 mg/ml) in absence and presence of BIL (1.3-50.2 μM). Measurements are done in 0.1 M PB (pH = 7.3) at 298 K.

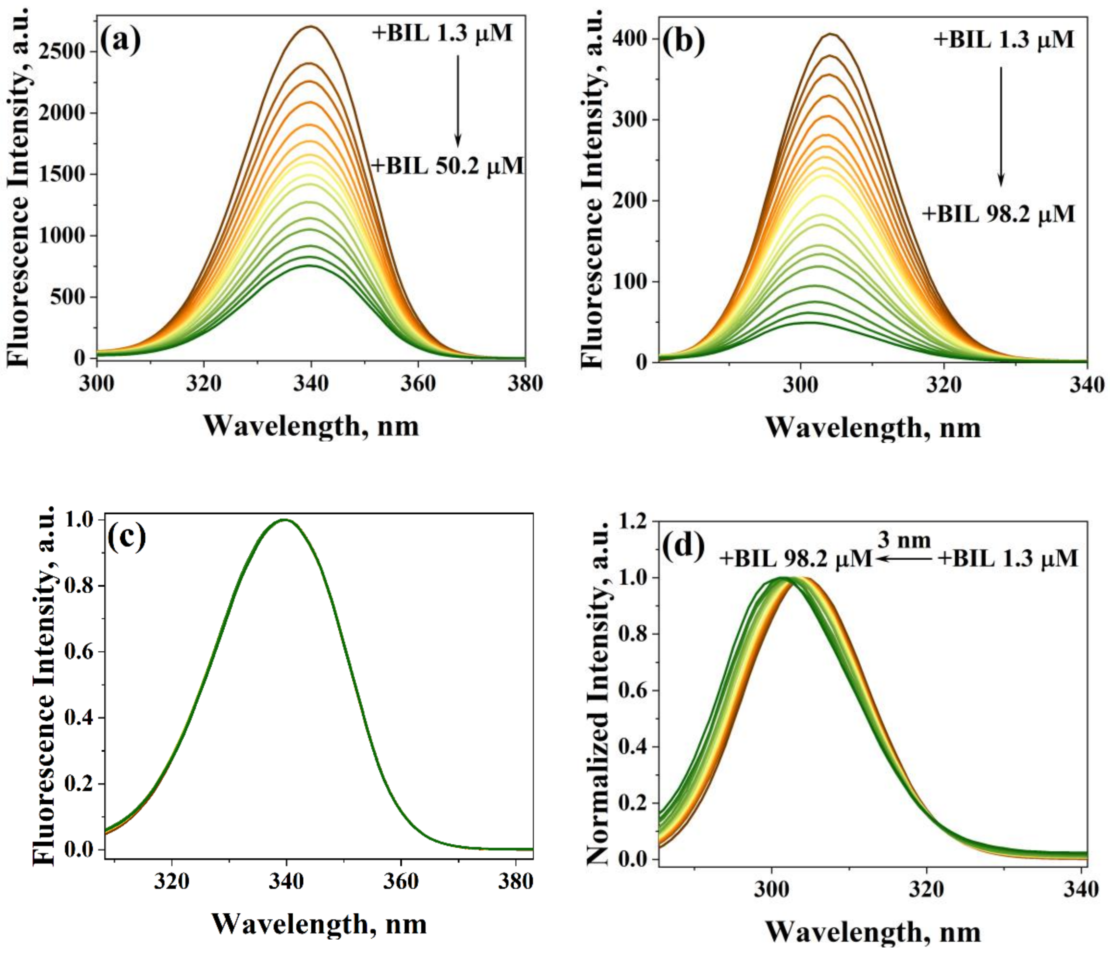


**Fig. S5.** (a) and (b) are synchronous fluorescence spectra of CAS protein (2 mg/ml) in absence and presence of BIL (1.3-50.2 μM) at Δλ = 60 nm and Δλ = 15 nm, respectively. (c) and (d) are corresponding normalized synchronous fluorescence spectra at Δλ = 60 nm and Δλ = 15 nm, respectively. Measurements are done in 0.1 M PB (pH = 7.3) at 298 K.

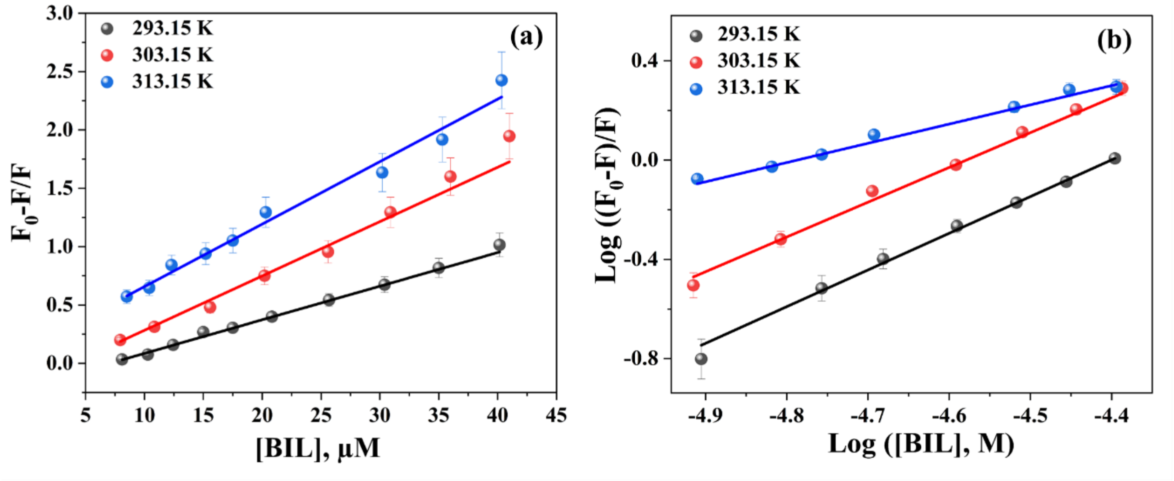


**Fig. S6.** (a) Modified stern Volmer plot and (b) Double logarithmic plot of CAS protein (2 mg/ml) in absence and presence of BIL (7-40 µM) in PB (pH = 7.3) at 293.15, 303.15 and 313.15 K.

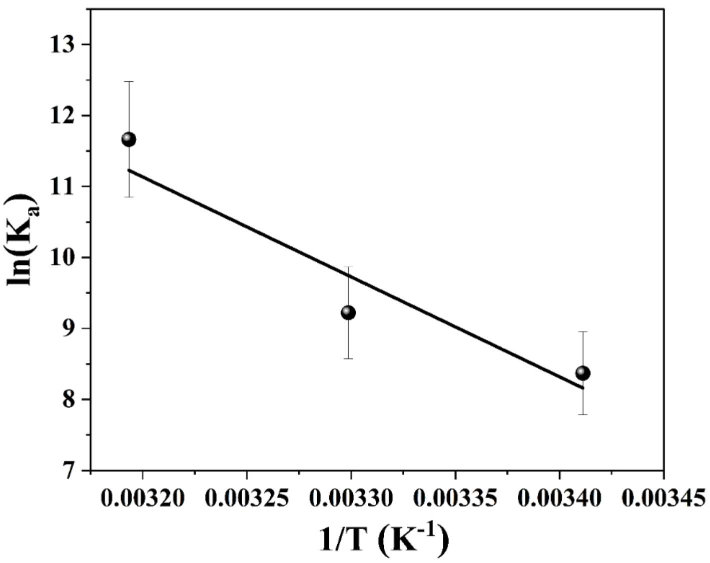


**Fig. S7.** Van't Hoff plot of CAS protein (2 mg/ml) in absence and presence of BIL (1.3-70 μM) in PB (pH = 7.3) at 293.15, 303.15 and 313.15 K.

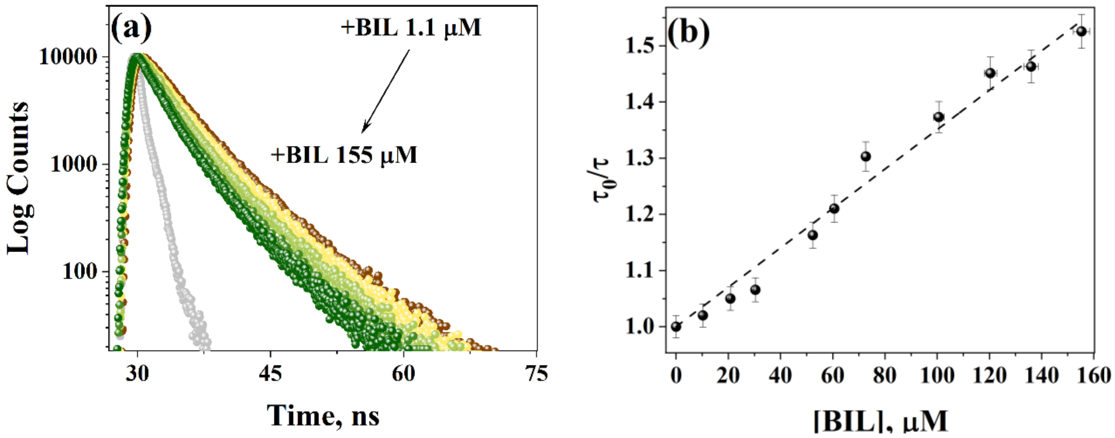


**Fig. S8.** (a) Fluorescence lifetime decay and (b) lifetime components of CAS (2 mg/ml) in absence and presence of BIL (1.1-155 μM) ($\lambda_{ex}$ = 291 nm and $\lambda_{em}$ = 335 nm). Measurements are done in 0.1 M PB (pH = 7.3) at 298 K.

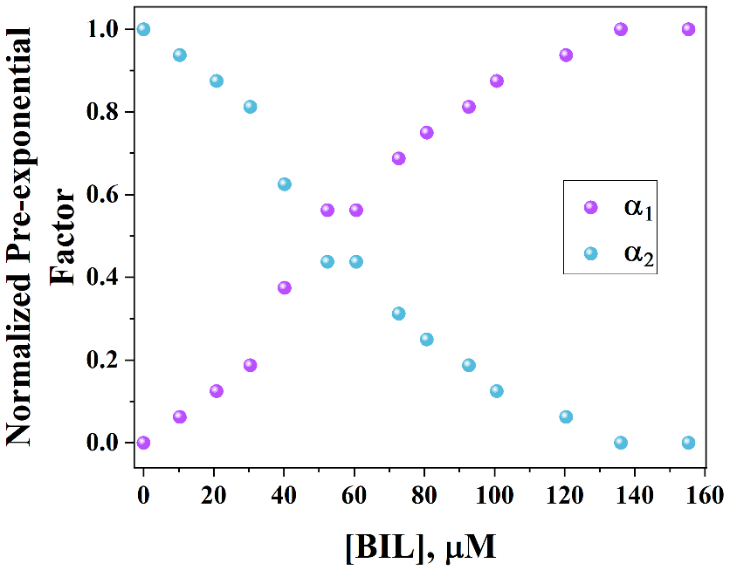


**Fig. S9.** Normalised pre-exponential factor of CAS as a function of increase in concentration of BIL (0-155.3 μM). Measurements are done in 0.1 M PB (pH = 7.3) at 298 K.

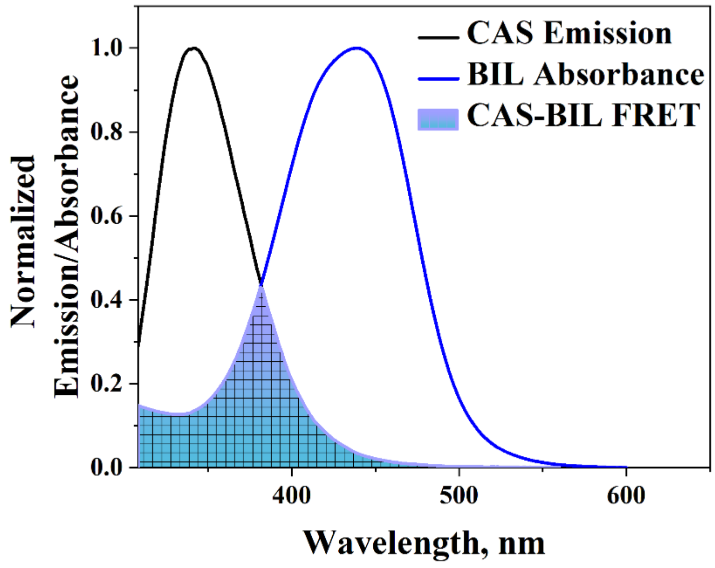


**Fig. S10.** Spectral overlap of CAS donor emission (Black) and acceptor BIL (Blue). The overlap between donor and acceptor is the FRET region.

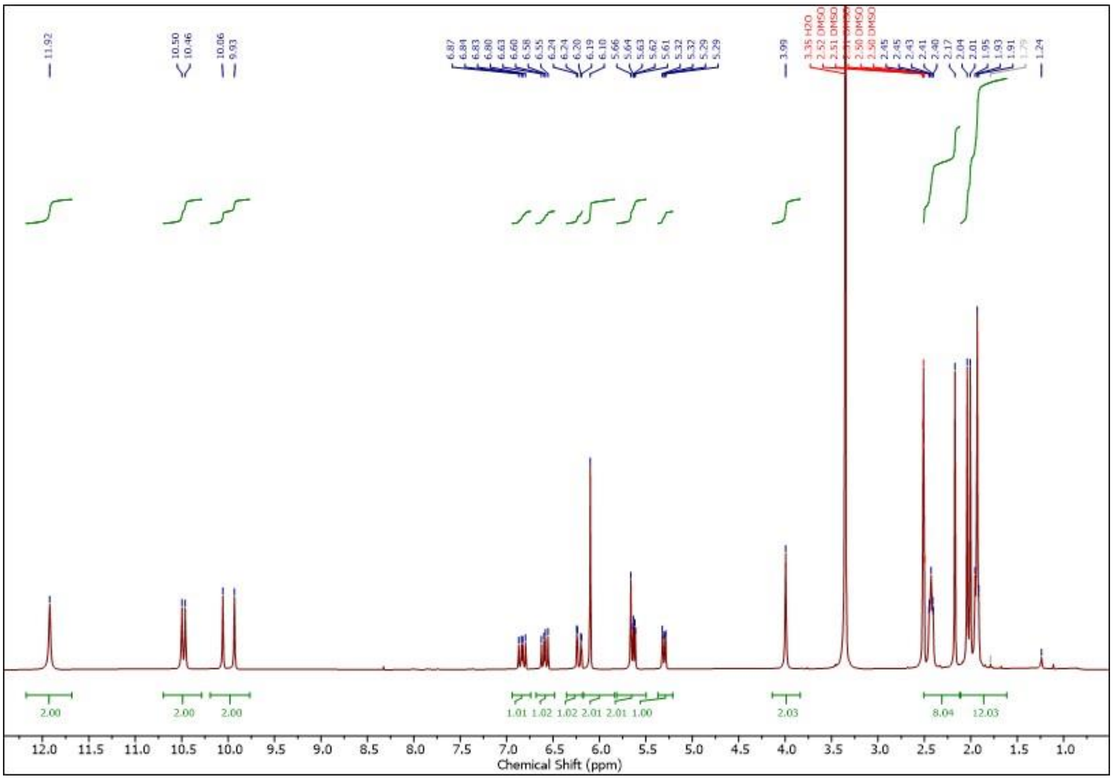

**Fig. S11.** $^1$H NMR spectrum (400 MHz) of BIL (10 mg/ml) in DMSO-$d_6$.

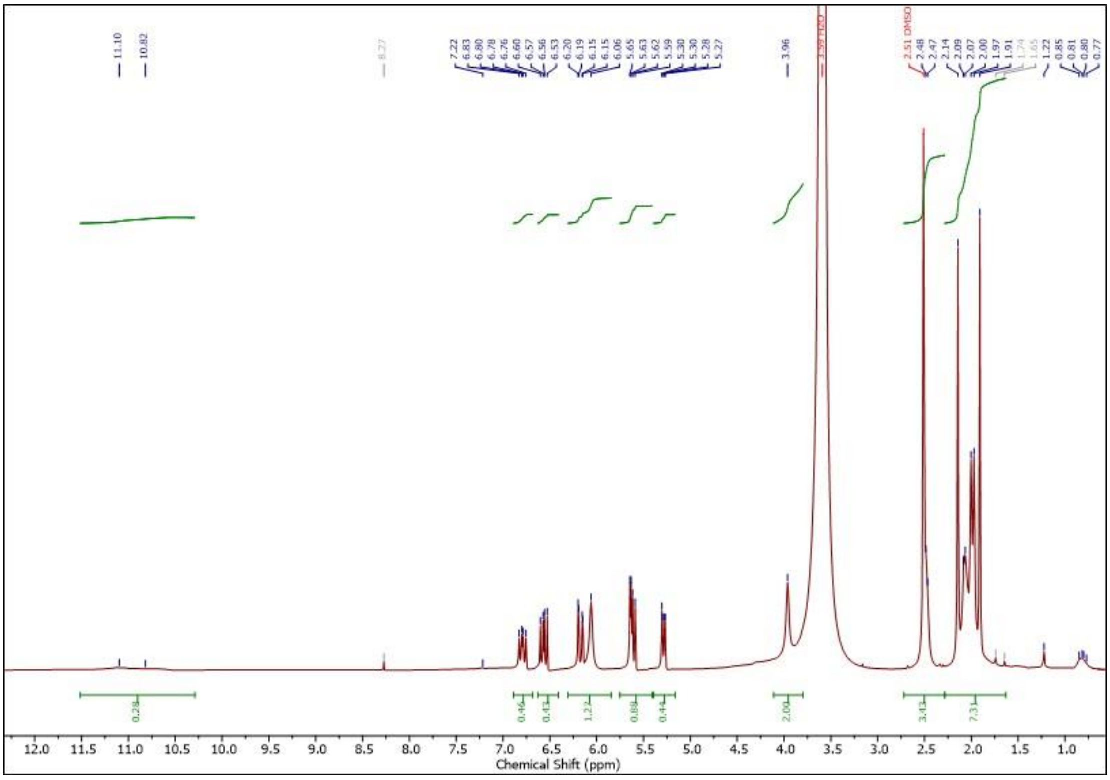


**Fig. S12.** $^{1}$H NMR spectrum (400 MHz) of BIL (10 mg/ml) + CAS (1 mg/ml) in DMSO-$d_6$.

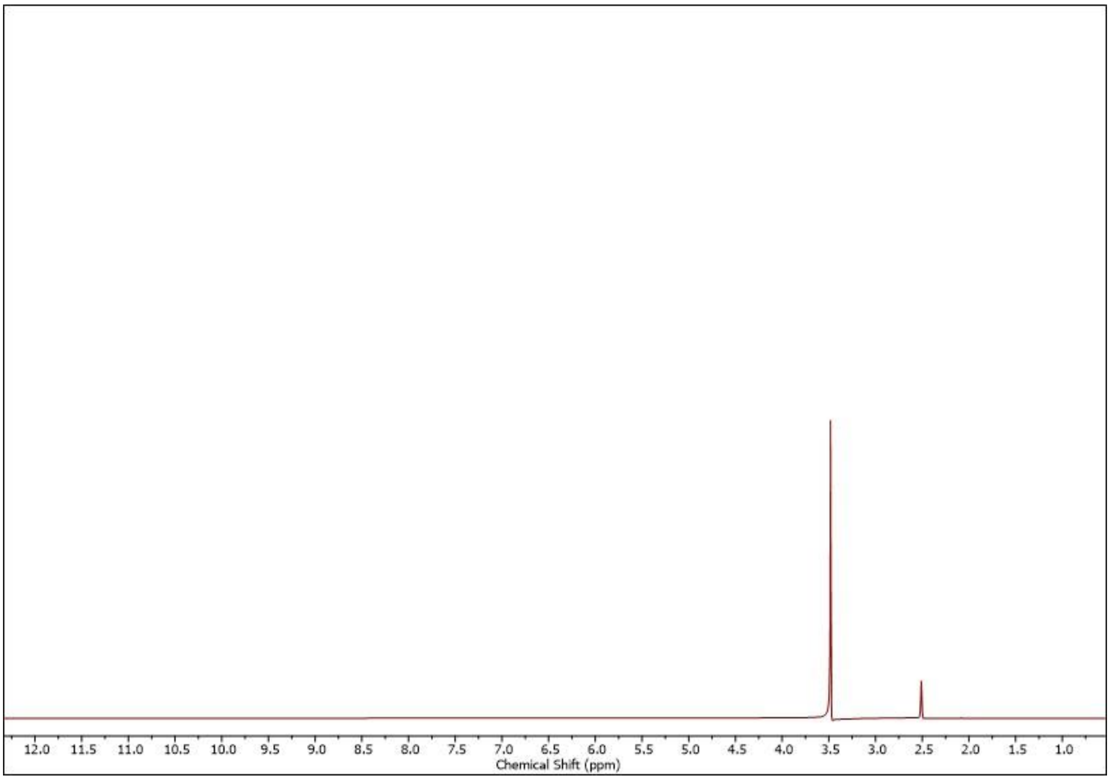


**Fig. S13.** $^{1}H$ NMR spectrum (400 MHz) of CAS (1 mg/ml) in DMSO-$d_6$.

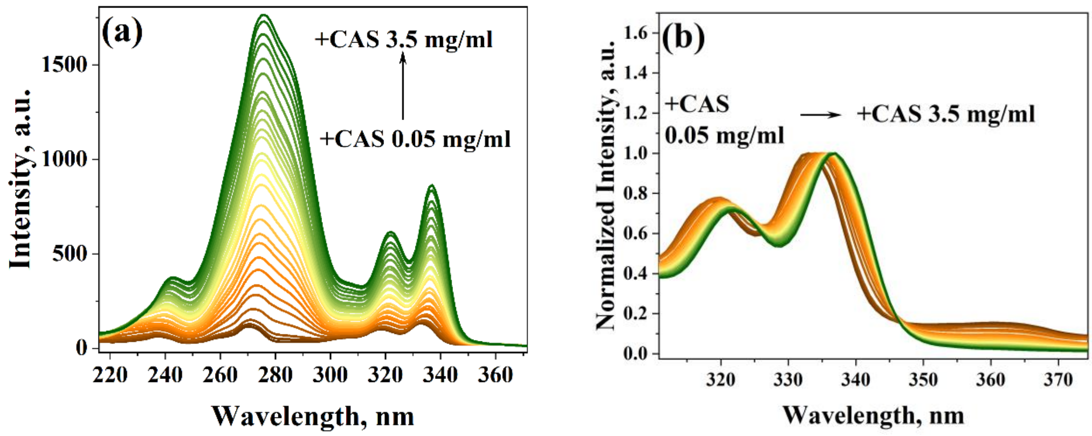


**Fig. S14.** (a) Excitation spectra ($\lambda_{em}$ = 390 nm) and (b) Normalised excitation spectra of pyrene (1.1 μM) in absence and presence of CAS (0.05-3.5 mg/ml). Measurements are done in 0.1 M PB (pH = 7.3) at 298 K.

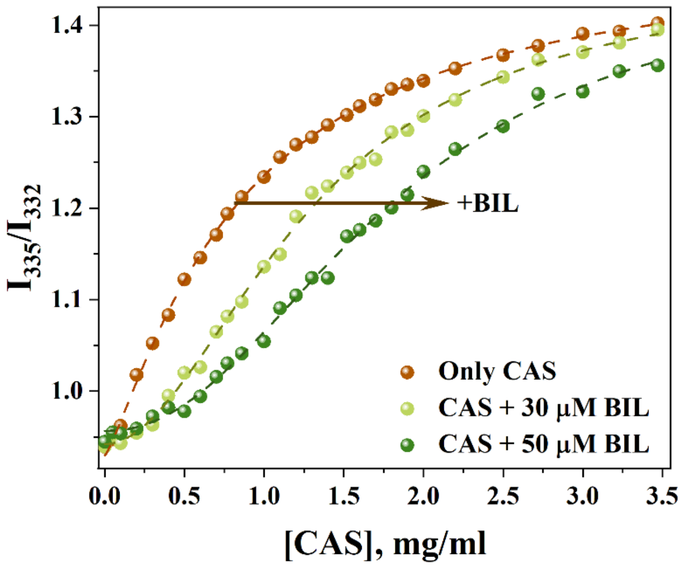


**Fig. S15.** $I_{335}/I_{332}$ band intensity ratio of pyrene as a function of the concentration of CAS protein in absence and presence of different BIL concentrations. Measurements are done in 0.1 M PB (pH = 7.3) at 298 K.

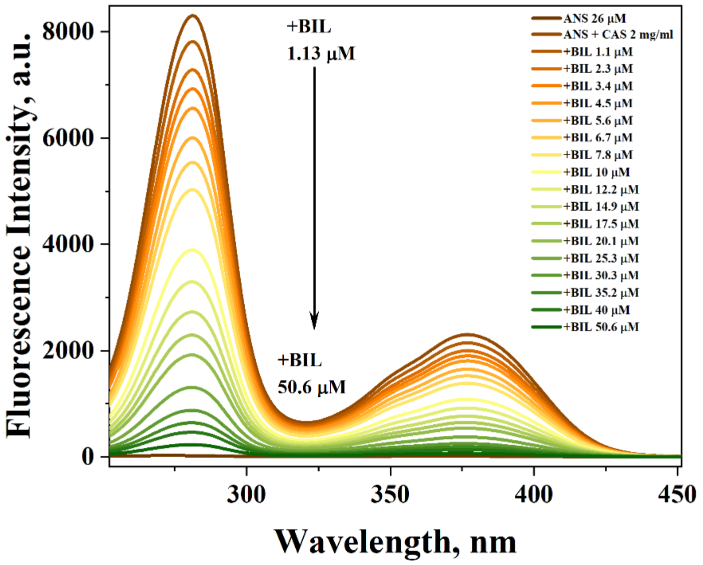


**Fig. S16.** Excitation spectra ($\lambda_{em}$ = 470 nm) of ANS (26 μM) with CAS (2 mg/ml) complex in absence and presence of BIL (1.13-50.6 mM). Measurements are done in 0.1 M PB (pH = 7.3) at 298 K.

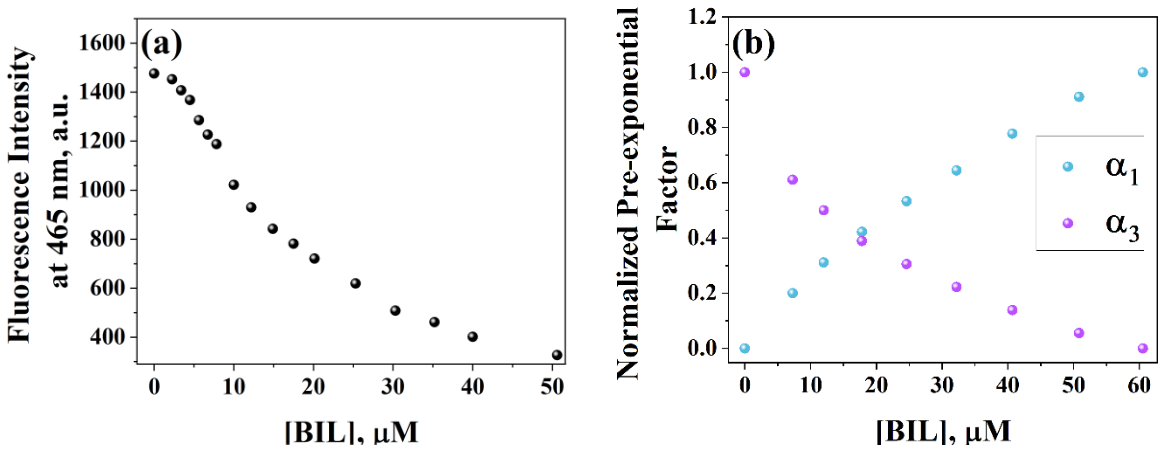


**Fig. S17.** (a) Emission intensity of ANS+CAS at wavelength maxima as a function of [BIL]. (b) Normalised pre-exponential factor of CAS bound ANS decays as a function of [BIL].

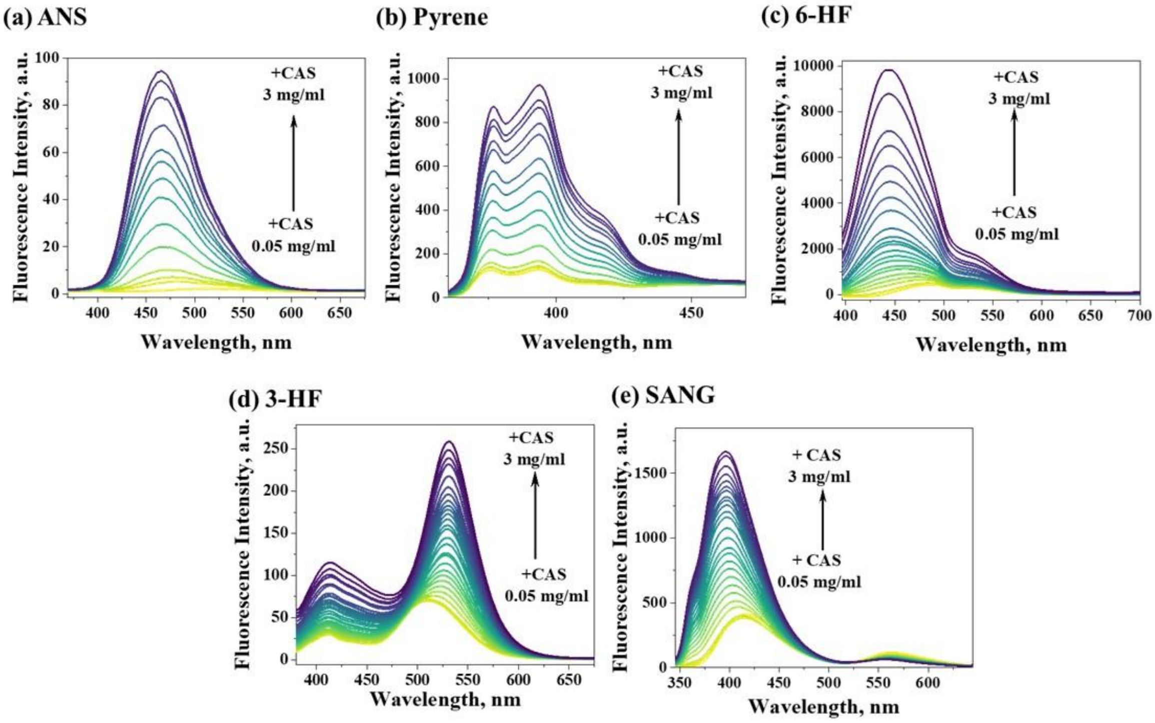


**Fig. S18.** Steady state emission spectra of (a) ANS ($\lambda_{ex}$ = 350 nm), (b) Pyrene ($\lambda_{ex}$ = 336 nm), (c) 6-HF ($\lambda_{ex}$ = 370 nm), (d) 3-HF ($\lambda_{ex}$ = 360 nm) and (e) SANG ($\lambda_{ex}$ = 330 nm) in presence of CAS. Measurements are done in 0.1 M PB (pH = 7.3) at 298 K.

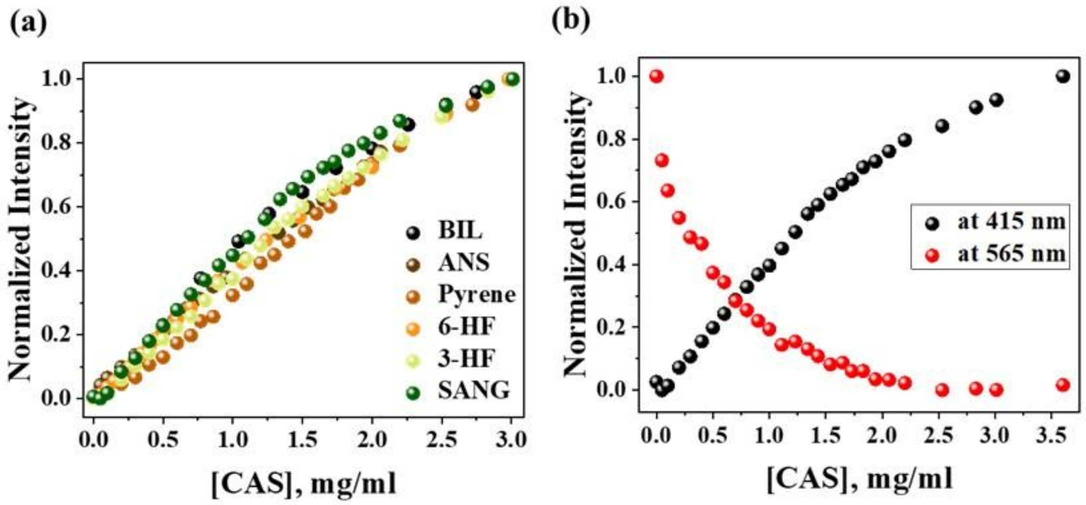


**Fig. S19.** (a) Normalized fluorescence intensity of the hydrophobic molecules as a function of different amounts of CAS. (b) Normalized fluorescence of SANG hydrophobic (alkanolamine, $\lambda_{em}$ = 415 nm) and hydrophilic (iminium, $\lambda_{em}$ = 565 nm) forms as a function of different amounts of CAS.

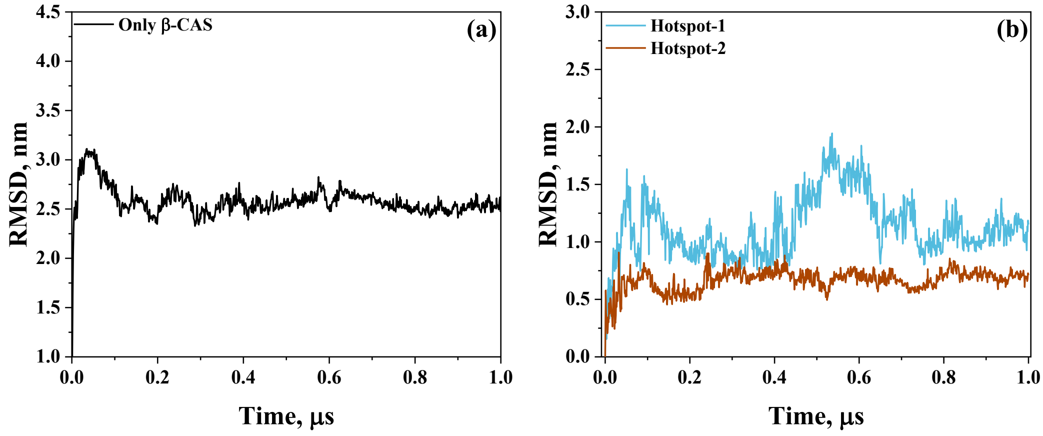


**Fig. S20.** (a) RMSD value variation for only β-CAS protein as a function of time. (b) RMSD value variation for β-CAS protein complex for 1 and 2 hotspots as a function of time. MD simulation was performed for 1 μs at 300 K temperature.

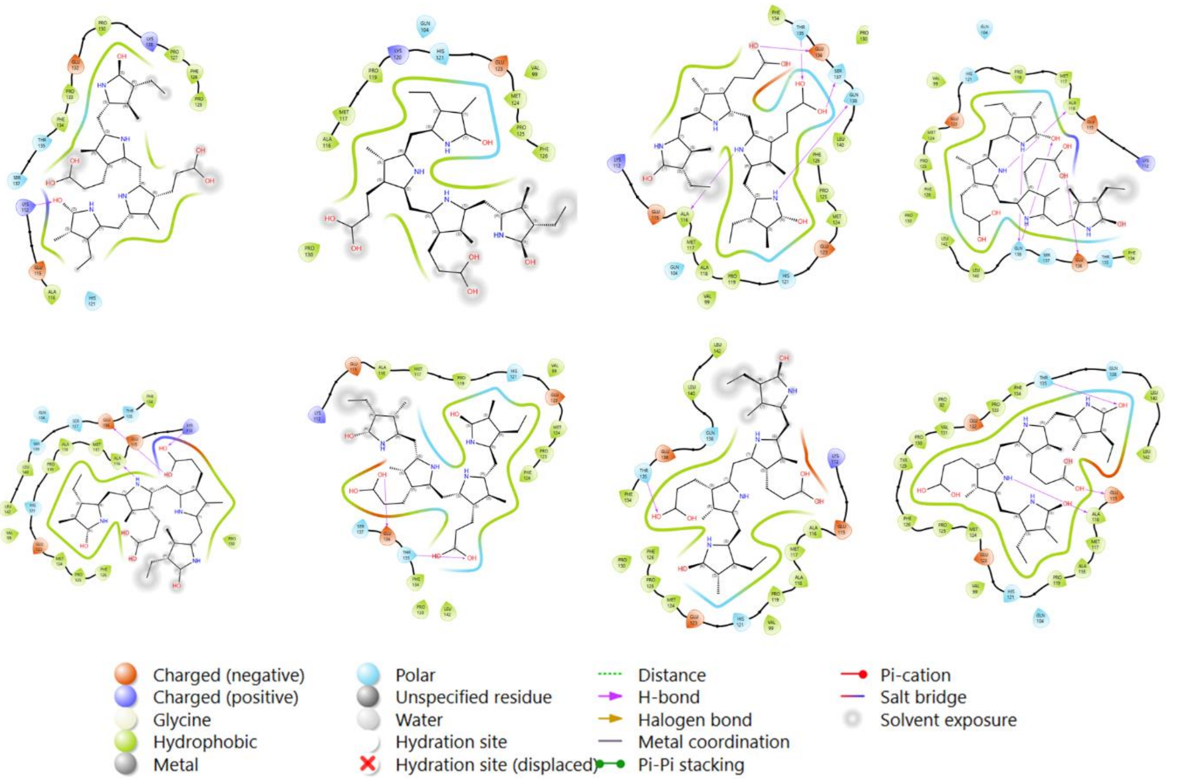


**Fig. S21.** Amino acid environment of BIL in complex-1 during the MD simulation trajectory from 0 to 700 ns (left to right starting from top panel) with 100 ns intervals.

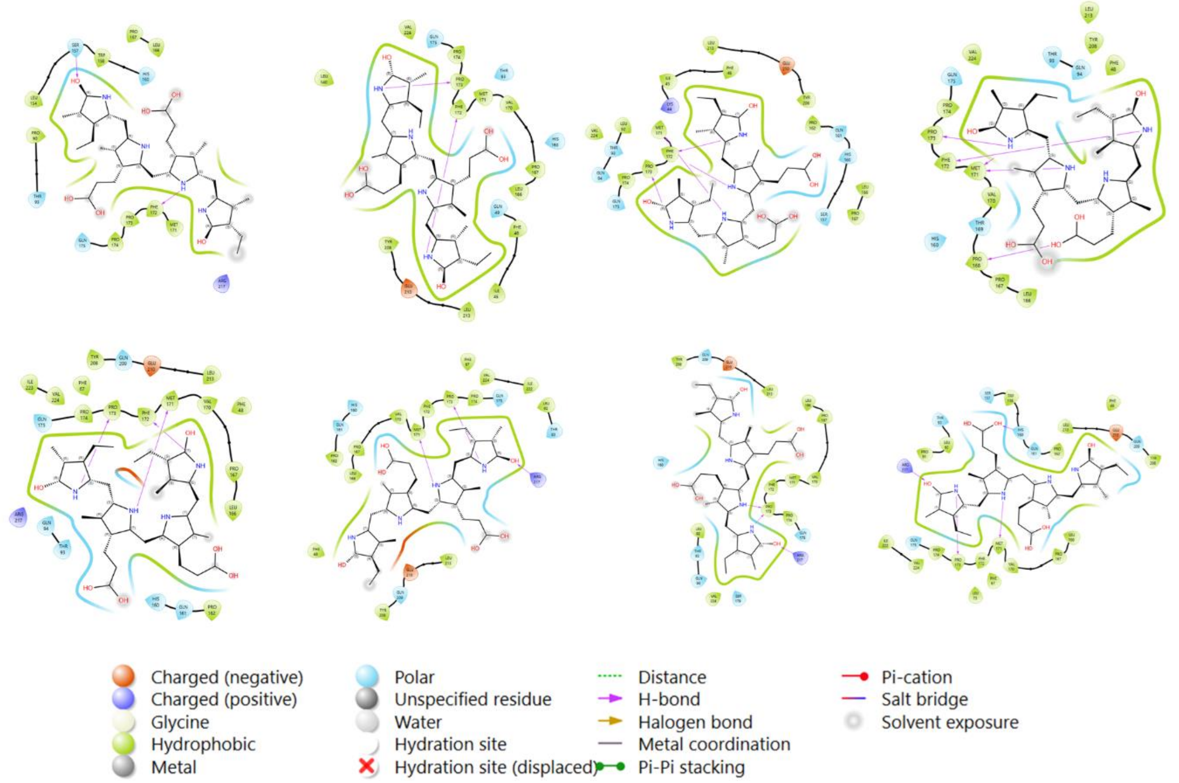


**Fig. S22.** Amino acid environment of BIL in complex-2 during the MD simulation trajectory from 0 to 700 ns (left to right starting from top panel) with 100 ns intervals.

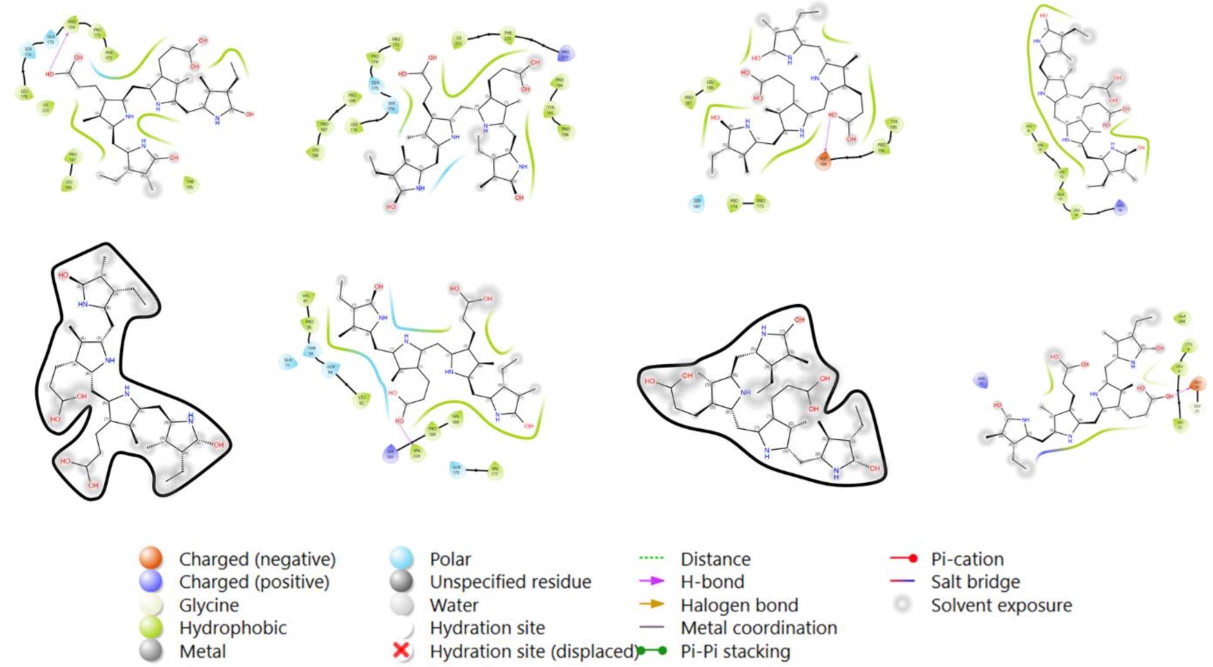


**Fig. S23.** Amino acid environment of BIL in complex-3 during the MD simulation trajectory from 0 to 700 ns (left to right starting from top panel) with 100 ns intervals.

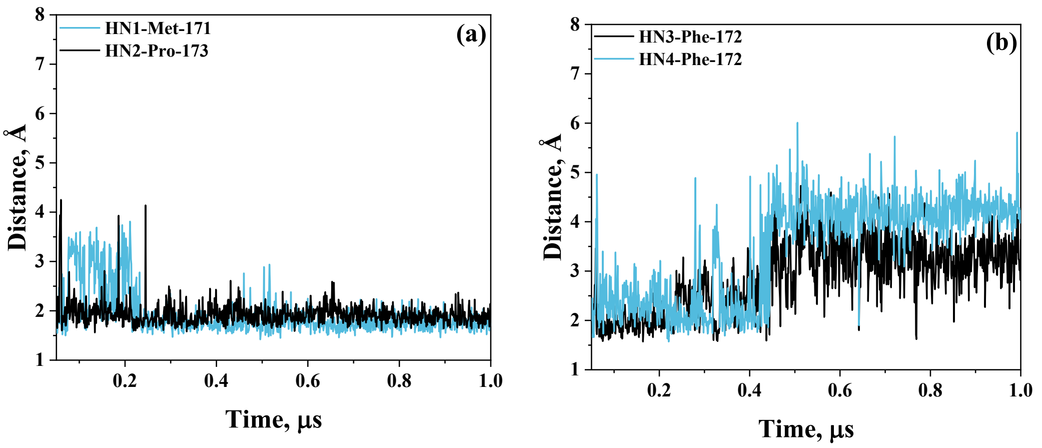


**Fig. S24.** Distance between hydrogen atom of four BIL nitrogen atoms and hotspot amino acids in complex-2.

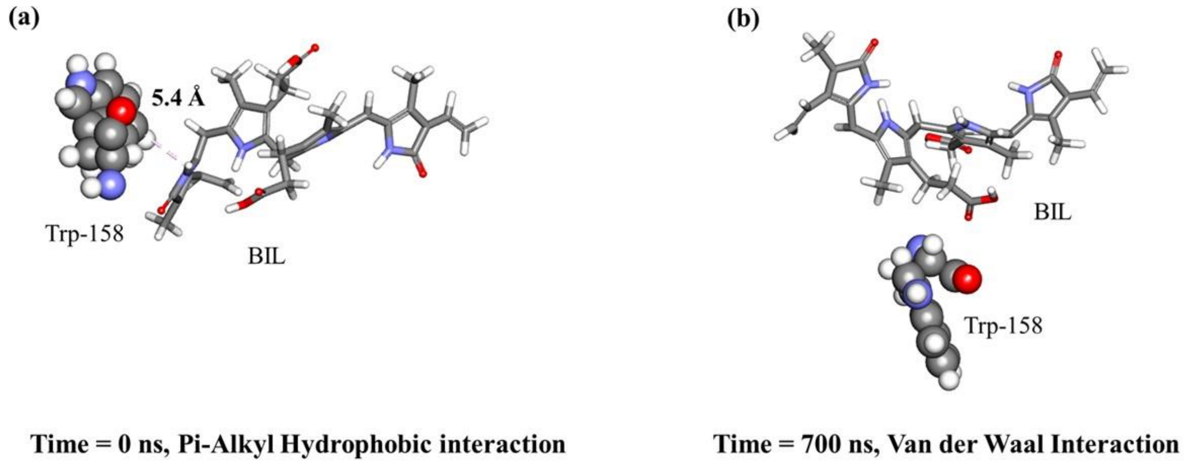


**Fig. S25.** Binding mode of Trp-158 and BIL at time (a) 0 ns and (b) 700 ns extracted from MD trajectory.

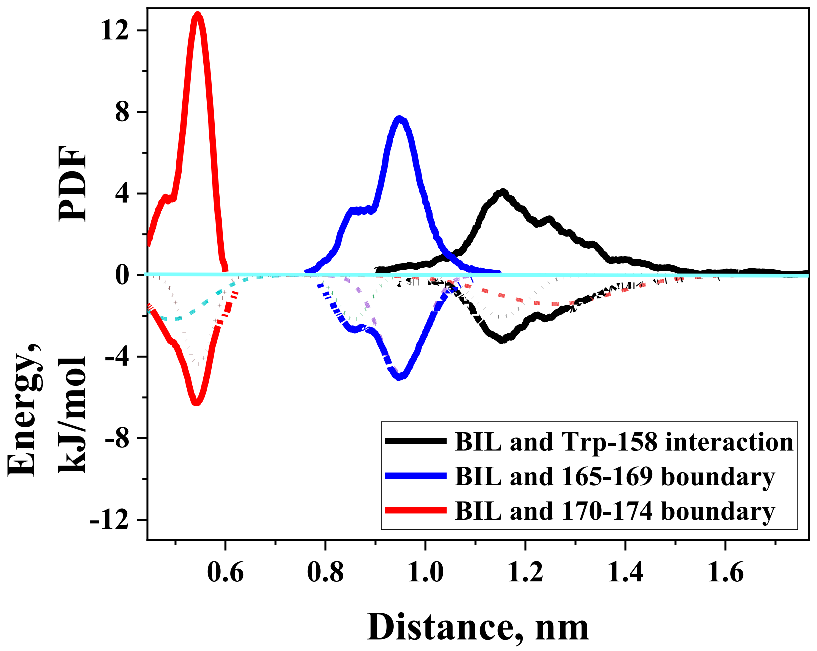


**Fig. S26.** Probability Distribution Function (PDF) and energy profiles ($-k_BTln(PDF)$) of Trp-158 and other hydrophobic interacting amino acids with BIL in complex 2. The energy profiles are further deconvoluted into short- and long-range interactions profiles.

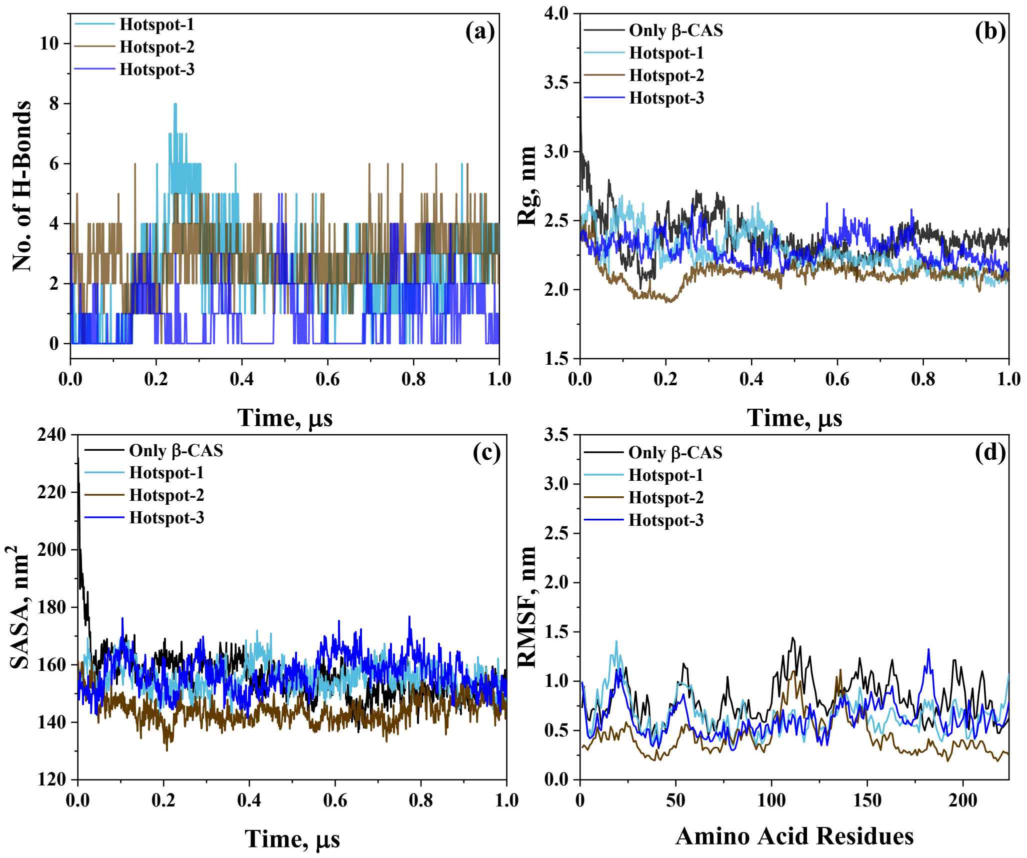


**Fig. S27.** MD simulation analysis for 1 μs time at 300K. (a) Number of H-bonds as a function of time. (b) Radius of gyration (Rg) as a function of time. (c) Solvent accessible surface area (SASA) as a function of time. (d) Root mean square fluctuation (RMSF) as a function of amino acid residues.

To analyse the protein structure and conformation, parameters such as radius of gyration (Rg), solvent accessible surface area (SASA) and root mean square fluctuation (RMSF) for the amino acid residues can be determined. (given in SI) **Fig. S27b** shows the comparison of Rg of only protein with the three complexes. The blank protein Rg value was maintained in a less fluctuating manner but has a higher average value (2.38 nm) than those of the complexes (2.27, 2.11 and 2.29 nm, respectively). Rg value for the complex-1 and 3 are in similar range, however, for the complex-2, it is lesser due to the dip as can be seen in the time region of 100-300 ns. On visualising the complex during this time, it was found that in this complex, the protein underwent a more folded structure bringing its two ends closer to each other and

causing the decrease in the Rg value. To analyse how the complex dynamics is affected by the solvent, we analysed SASA for the complexes and compared with the blank protein (**Fig. S27c**). The average area of the protein exposed to the solvent was 154.3 $nm^2$ and in case of complexes it was found to be 155.5, 144 and 156.8 $nm^2$, respectively. The average values for the complex-1 and complex-3 are more than that of blank protein, however surprisingly, for the complex-2, the SASA value is much lower with a comparatively very high difference which is also visible in the SASA plot. This much lowering of SASA clearly supports the folding mechanism which was also the reason for the decrease in the Rg value for complex-2. We also analysed the RMSF for the amino acid positions in absence and presence of ligand. For blank protein, the average value of RMSF is 0.81 nm whereas for complexes 1, 2 and 3 are 0.65, 0.44 and 0.63 nm, respectively with again distinctly least fluctuation in that for hotspot-2. These results suggest a folded protein conformation is present that is the resultant of the strong H-bonding interaction between BIL and protein at hotspot-2.

Additionally, the fluctuation in the amino acids (**Fig. S27d**) which were involved in the H-bonding, Met-171, Phe-172 and Pro-173 can be determined. It can be seen that the fluctuation for these amino acids is minimal during the simulation for the complex-2. For blank protein, the average fluctuation in SASA for these amino acids are 0.29, 1.22 and 0.45 $nm^2$, respectively. Whereas in presence of ligand at hotspot-1, these values change to: 0.22, 1, 0.48 $nm^2$, at hotspot-2: 0.19, 0.35, 0.06 $nm^2$ and at hotspot-3, 0.31, 0.84, 0.17 $nm^2$.

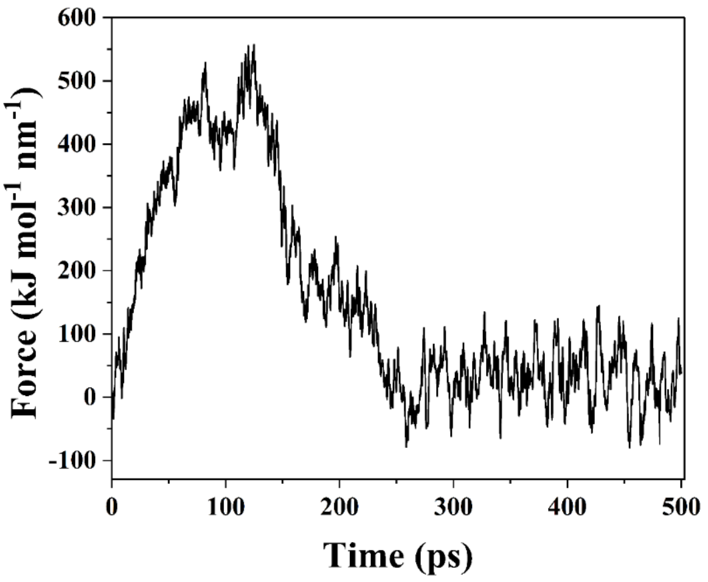


**Fig. S28.** Plot corresponding to dissociation pathway of force versus time over 500 ps.

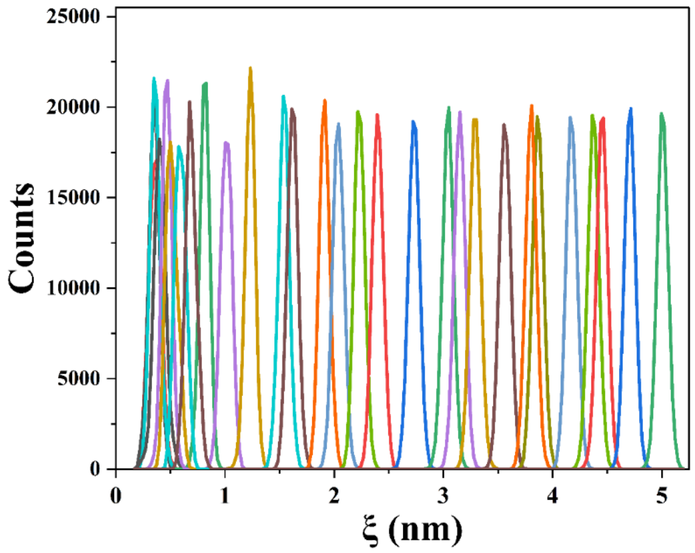


**Fig. S29**. Histograms of the pulling in the z-axis from ξ = 0.14 nm to ξ = 5.12 nm.

# REFERENCES


(1) Jacobsen, J.; Wennberg, R. P. Determination of unbound bilirubin in the serum of newborns. *Clin. Chem.* **1974**, *20* (7), 783-789.
(2) Suo, Z.; Sun, Q.; Yang, H.; Tang, P.; Gan, R.; Xiong, X.; Li, H. Combined spectroscopy methods and molecular simulations for the binding properties of trametinib to human serum albumin. *RSC Adv.* **2018**, *8* (9), 4742-4749.
(3) Hill, A. V. The possible effects of the aggregation of the molecules of hemoglobin on its dissociation curves. *j. physiol.* **1910**, *40*, iv-vii.
(4) Lakowicz, J. R.; Lakowicz, J. R. Instrumentation for fluorescence spectroscopy. *Principles of fluorescence spectroscopy* **1999**, 25-61.
(5) Li, D.; Zhu, J.; Jin, J.; Yao, X. Studies on the binding of nevadensin to human serum albumin by molecular spectroscopy and modeling. *J. Mol. Struct.* **2007**, *846* (1-3), 34-41.
(6) Tanwar, A. S.; Parui, R.; Garai, R.; Chanu, M. A.; Iyer, P. K. Dual "Static and Dynamic" fluorescence quenching mechanisms based detection of TNT via a cationic conjugated polymer. *ACS Meas. Sci. Au* **2021**, *2* (1), 23-30.
(7) Moeiniafshari, A.-A.; Zarrabi, A.; Bordbar, A.-K. Exploring the interaction of naringenin with bovine beta-casein nanoparticles using spectroscopy. *Food Hydrocolloids* **2015**, *51*, 1-6.
(8) Willcott, M. R. MestRe nova. ACS Publications: 2009.
(9) Frisch, M. J.; Trucks, G. W.; Schlegel, H. B.; Scuseria, G. E.; Robb, M. A.; Cheeseman, J. R.; Scalmani, G.; Barone, V.; Petersson, G. A.; Nakatsuji, H.; et al. Gaussian 16 Rev. C.01. **2016**.
(10) Baek, M.; DiMaio, F.; Anishchenko, I.; Dauparas, J.; Ovchinnikov, S.; Lee, G. R.; Wang, J.; Cong, Q.; Kinch, L. N.; Schaeffer, R. D. Accurate prediction of protein structures and interactions using a three-track neural network. *Science* **2021**, *373* (6557), 871-876.
(11) Abraham, M. J.; Murtola, T.; Schulz, R.; Páll, S.; Smith, J. C.; Hess, B.; Lindahl, E. GROMACS: High performance molecular simulations through multi-level parallelism from laptops to supercomputers. *SoftwareX* **2015**, *1*, 19-25.
(12) Huang, J.; Rauscher, S.; Nawrocki, G.; Ran, T.; Feig, M.; De Groot, B. L.; Grubmüller, H.; MacKerell Jr, A. D. CHARMM36m: an improved force field for folded and intrinsically disordered proteins. *Nat. Methods* **2017**, *14* (1), 71-73.
(13) Essmann, U.; Perera, L.; Berkowitz, M. L.; Darden, T.; Lee, H.; Pedersen, L. G. A smooth particle mesh Ewald method. *J. Chem. Phys.* **1995**, *103* (19), 8577-8593.
(14) Berendsen, H. J.; Postma, J. v.; Van Gunsteren, W. F.; DiNola, A.; Haak, J. R. Molecular dynamics with coupling to an external bath. *J. Chem. Phys.* **1984**, *81* (8), 3684-3690.
(15) Gowers, R. J.; Linke, M.; Barnoud, J.; Reddy, T. J. E.; Melo, M. N.; Seyler, S. L.; Domanski, J.; Dotson, D. L.; Buchoux, S.; Kenney, I. M. MDAnalysis: a Python package for the rapid analysis of molecular dynamics simulations. **2019**.
(16) Eberhardt, J.; Santos-Martins, D.; Tillack, A. F.; Forli, S. AutoDock Vina 1.2. 0: New docking methods, expanded force field, and python bindings. *J. Chem. Inf. Model.* **2021**, *61* (8), 3891-3898.
(17) Bugnon, M.; Goullieux, M.; Röhrig, U. F.; Perez, M. A.; Daina, A.; Michielin, O.; Zoete, V. SwissParam 2023: a modern web-based tool for efficient small molecule parametrization. *J. Chem. Inf. Model.* **2023**, *63* (21), 6469-6475.
(18) Zoete, V.; Cuendet, M. A.; Grosdidier, A.; Michielin, O. SwissParam: a fast force field generation tool for small organic molecules. *J. Comput. Chem.* **2011**, *32* (11), 2359-2368.
(19) Schrödinger Release 2024-4: Maestro, Schrödinger. **2024**, (Schrödinger Release 2024-4).
(20) Roux, B. The calculation of the potential of mean force using computer simulations. *Comput. Phys. Commun.* **1995**, *91* (1-3), 275-282.
(21) Kumar, S.; Rosenberg, J. M.; Bouzida, D.; Swendsen, R. H.; Kollman, P. A. The weighted histogram analysis method for free-energy calculations on biomolecules. I. The method. *J. Comput. Chem.* **1992**, *13* (8), 1011-1021.

(22) Marques, M. A.; Ullrich, C. A.; Nogueira, F.; Rubio, A.; Burke, K.; Gross, E. K. *Time-dependent density functional theory*; Springer, 2006.
(23) Tirado-Rives, J.; Jorgensen, W. L. Performance of B3LYP density functional methods for a large set of organic molecules. *J. Chem. Theory Comput.* **2008**, *4* (2), 297-306.
(24) Cossi, M.; Scalmani, G.; Rega, N.; Barone, V. New developments in the polarizable continuum model for quantum mechanical and classical calculations on molecules in solution. *J. Chem. Phys.* **2002**, *117* (1), 43-54.
